# Isolation of Anti-Diabetic Active Compounds from Benincasae Exocarpium and Development of Simultaneous Analysis by HPLC-PDA

**DOI:** 10.3390/molecules27010009

**Published:** 2021-12-21

**Authors:** Hye Sung Ryu, Suk Jin Lee, Wan Kyunn Whang

**Affiliations:** Department of Global Innovative Drug, Graduate School of Chung-Ang University, College of Pharmacy, Chung-Ang University, Heukseok-dong, Dongjak-gu, Seoul 151-756, Korea; hyesung0215@gmail.com (H.S.R.); sukjin37@cau.ac.kr (S.J.L.)

**Keywords:** *Benincasa cerifera* Savi., Benincasae Exocarpium, type 2 diabetes, flavonoid, simultaneous analysis

## Abstract

Diabetes is a chronic metabolic disease that is a constant problem. Previous studies have reported that *Benincasa cerifera* Savi. extracts are effective in treating diabetes and its complications. Benincasae Exocarpium (BE) is a fruit peel of *B. cerifera* that has been reported to be used for the prevention and treatment of metabolic diseases such as hyperglycemia, obesity, and hyperlipidemia. However, there are not enough studies on the compounds and bioassays to support the efficacy of BE. The inhibitory activity of the BE extracts and fractions against advanced glycation end-products (AGE) formation and α-glucosidase activity was evaluated. These assays are relevant for the treatment of type 2 diabetes and its complications. Based on these results, compounds **1**–**11** were isolated through bioassay-guided isolation. In addition, we developed a high-performance liquid chromatography (HPLC) method that can simultaneously analyze these 11 compounds. Activity evaluation of the compounds was also conducted, and eight compounds exhibited significant activity. Among these, flavonoid compounds showed strong activity. A quantitative evaluation of eight bioactive compounds (**2**, **5**–**11**) was conducted. In conclusion, this study demonstrated the potential of BE for prevention and treatment of type 2 diabetes and its complications.

## 1. Introduction

The number of diabetic patients is steadily increasing worldwide, and type 2 diabetes, especially among young people such as children and adolescents, is becoming a problem [1]. The causes of type 2 diabetes include environmental exposure and genetic factors [2]. Early diagnosis and management of risk factors are important because diabetes causes various serious complications. Blood sugar management is important for the prevention and management of type 2 diabetes [3], and agents with α-glucosidase inhibitory activity are used as oral hypoglycemic drugs [4]. In the state of hyperglycemia, a sugar-derived substances called advanced glycation end-products (AGEs) are actively produced and accumulate in the blood and tissue [5,6]. AGEs are a heterogeneous group of molecules formed by the Maillard reaction [7]. The reaction is a non-enzymatic reaction of reducing sugars with free amino groups of proteins, lipids, and nucleic acids [5]. Methylglyoxal (MGO) is one of the most reactive AGE precursors [8]. During aging and diabetes, increasing amounts of AGE-modified proteins can be detected. In other words, they are involved in the development of degenerative diseases such as diabetes [9]. Therefore, controlling the formation of AGEs is important for the prevention and treatment of diabetes and diabetic complications. Agents that inhibit or reduce AGE formation include aminoguanidine, pyridoxamine, and OPB-9195. Aminoguanidine has been reported to be toxic during clinical evaluation [6]. Therefore, it is necessary to identify a safe anti-glycation agent. To find novel synthetic AGE inhibitors, scientists are focusing on researching anti-glycation compounds from natural products [10].

*Benincasa cerifer*a Savi. belongs to the Cucurbitaceae family and is commonly called ash gourd, wax gourd, winter melon, Chinese watermelon, and donggua [11]. It is extensively cultivated in southern China, southeast Asia [12], and Korea. In India, the fruit is traditionally eaten as a fruit and used as a medicinal herb. In addition, it is used as a nutrient, tonic, diuretic, aphrodisiac, and styptic in various diseases and disorders such as asthma, bronchitis, insanity, epilepsy, dry cough, fever, and syphilis [13]. According to previous studies, B. cerifera extract is effective in antioxidant, treatment of diabetes [14] and its complications [15], anti-ulcer activity [16], and suppression of allergic inflammation [17]. The compounds isolated from *B. cerifera* include multiflorenol, isovitexin, astillbin, catechin, naringenin, 5-Hydroxymethylfurfural, gallic acid, rutin, and quercetin [18,19,20,21].

Benincasae Exocarpium (BE) is a fruit peel of *B. cerifera* and has been used in traditional Chinese medicine for the treatment and prevention of metabolic diseases such as hyperglycemia and obesity [22]. However, existing specific scientific experiments are insufficient. In addition, due to the lack of research on the compounds of BE, an appropriate content standard cannot be presented. Therefore, quality control standards have not yet been established. For these reasons, there are limitations to the development of medicines and health functional foods using BE. In this study, scientific anti-diabetes active assays such as AGE formation in bovine serum albumin (BSA)-glucose and BSA-methylglyoxal (MGO) systems and α-glucosidase were conducted on BE extracts and fractions. Eleven compounds were isolated by bioassay-guided isolation. Activity experiments on the compounds were also performed. In addition, through the development of an optimized extraction process and a multi-component simultaneous content analysis method using high-performance liquid chromatography-photodiode array detector (HPLC-PDA), the components of BE were profiled. Through experiments, the criteria for BE content were set. As a result, we proposed quality control standards for BE for the first time.

## 2. Results and Discussion

### 2.1. Identification of Compounds ***1**–**11*** Isolated from Benincasae Exocarpium

Isolated compounds **1**–**11** were identified as *p*-hydroxybenzoic acid (**1**), protocatechuic acid (**2**), isovanillin (**3**), 5-hydroxymethylfurfural (**4**), isovitexin (**5**), vitexin (**6**), orientin (**7**), gallic acid (**8**), caffeic acid (**9**), vitexin-2″-*O*-rhamnoside (**10**), and vitexin-4″-*O*-glucoside (**11**) using NMR (^1^H and ^13^C) spectroscopy and ESI-QTOF-MS/MS and through comparison with previous studies (Supplementary Materials, Figures S1–S33) [23,24,25,26,27,28,29,30,31,32,33].

#### 2.1.1. *p*-Hydroxybenzoic Acid

C_7_H_6_O_3_; ESI-QTOF-MS/MS *m/z*: 137.0235 [M − H]^−^, ^1^H-NMR (600 MHz, CD_3_OD-*d_4_*) δ: 7.87 (2H, td, *J* = 5.9, 3.4 Hz, H-3, 5); 6.81 (2H, dd, *J* = 2.4 Hz, H-2, 6), ^13^C-NMR (150 MHz, CD_3_OD-*d_4_*) δ: 170.10 (C-7), 163.33 (C-4), 133.00 (C-3, 5), 122.68 (C-1), 116.03 (C-2, 6).

#### 2.1.2. Protocatechuic Acid

C_7_H_6_O_4_; ESI-QTOF-MS/MS *m/z*: 153.0182 [M − H]^−^, ^1^H-NMR (600 MHz, CD_3_OD-*d_4_*) δ: 7.43–7.41 (2H, m, CH, CH), 6.79 (1H, d, *J* = 8.3 Hz, CH); ^1^^3^C-NMR (150 MHz, CD_3_OD-*d_4_*) δ: 170.23 (C-7), 151.50 (C-4), 146.03 (C-3), 123.89 (C-1), 123.11 (C-6), 117.72 (C-2), 115.76 (C-5).

#### 2.1.3. Isovanillin

C_8_H_8_O_3_; ESI-QTOF-MS/MS *m/z*: 151.1472 [M − H]^−^, ^1^H-NMR (600 MHz, CDCl_3_-*d*) δ: 9.84 (1H, s, CHO), 7.44–7.42 (2H, m, H-2, 6), 6.97 (1H, d, *J* = 8.3 Hz, H-5), 5.78 (1H, s, H-3), 3.99 (3H, s, OCH_3_), 1.63 (1H, s, H-1); ^13^C-NMR (150 MHz, CDCl_3_-d) δ: 191.00 (CHO), 151.76 (C-5), 146.13 (C-2), 130.68 (C-1), 124.51 (C-4), 114.09 (C-3), 110.17 (C-6), 56.18 (OCH_3_).

#### 2.1.4. 5-Hydroxymethylfurfural

C_6_H_6_O_3_; ESI-QTOF-MS/MS *m/z*: 125.0236 [M − H]^−^, ^1^H-NMR (600 MHz, CDCl_3_-*d*) δ: 9.58 (1H, s, H-1), 7.21 (1H, d, *J* = 3.4 Hz, H-3), 6.51 (1H, d, *J* = 3.4 Hz, H-4), 4.71 (2H, s, H-6); ^13^C-NMR (150 MHz, CDCl_3_-d) δ: 177.06 (C-1), 160.54 (C-2), 152.36 (C-5), 122.71 (C-3), 109.96 (C-4), 57.61 (C-6).

#### 2.1.5. Isovitexin

C_21_H_20_O_10_; ESI-QTOF-MS/MS *m/z*: 431.0967 [M − H]^−^, ^1^H-NMR (600 MHz, DMSO-*d_6_*) δ 7.92 (2H, d, *J* = 8.3 Hz, H-2′, 6′), 6.91 (2H, d, *J* = 9.0 Hz, H-3′, 5′), 6.77 (1H, s, H-3), 6.50 (1H, s, H-8), 4.87 (2H, d, *J* = 5.5 Hz, H-1″, 2″), 4.57 (1H. d, *J* = 9.6 Hz, H-3″), 4.03 (1H, t, *J* = 9.0 Hz, H-6″), 3.67 (1H, d, *J* = 11.0 Hz, H-5″), 3.23–3.09 (2H, m, H-3″, 4″); ^13^C-NMR (150 MHz, DMSO-*d_6_*) δ: 181.96 (C-4), 163.52 (C-2), 163.32 (C-7), 161.18 (C-4′), 160.67 (C-5), 156.23 (C-9), 128.48 (C-2′, 6′), 121.11 (C-1′), 115.99 (C-3′, 5′), 108.89 (C-6), 103.39 (C-10), 102.79 (C-3), 93.63 (C-8), 81.58 (C-5″), 78.94 (C-3″), 73.05 (C-1″), 70.61 (C-2″), 70.22 (C-4″), 61.48 (C-6″).

#### 2.1.6. Vitexin

C_21_H_20_O_10_; ESI-QTOF-MS/MS *m/z*: 431.0211 [M − H]^−^, ^1^H-NMR (600 MHz, DMSO-*d_6_*) δ: 7.70(2H, d, *J* = 8.2 Hz, H-2′, 6′), 6.84 (2H, d, *J* = 8.0 Hz, H-3′, 5′), 6.47 (1H, s, H-3), 6.30 (1H, s, H-8), 4.74 (1H, d, *J* = 8.9 Hz, H-1″), 4.04 (1H, t, *J* = 8.1 Hz, H-2″), 3.69 (1H, dd, *J* = 11.8, 1.7 Hz, H-6b″), 3.64 (1H, dd, *J* = 13.5, 5.8 Hz, H-6a″), 3.55 (1H, m, H-4″), 3.53 (1H, m, H-3″), 3.51 (1H, m, H-5″); ^13^C-NMR (150 MHz, DMSO-*d_6_*) δ: 181.42 (C-4), 164.11 (C-2), 163.42 (C-7), 161.95 (C-4′), 159.14 (C-5), 157.21 (C-9), 128..74 (C-2′, 6′), 121.42 (C-1′), 115.24 (C-3′, 5′), 107.61 (C-6), 103.25 (C-10), 102.88 (C-3), 94.27 (C-8), 80.45 (C-5″), 78.64 (C-3″), 74.41 (C-1″), 71.11 (C-2″), 69.10 (C-4″), 60.00 (C-6″).

#### 2.1.7. Orientin

C_21_H_20_O_11_; ESI-QTOF-MS/MS *m/z*: 447.1042 [M − H]^−^, ^1^H-NMR (600 MHz, DMSO-*d_6_*) δ: 13.07 (1H, s, OH-5), 10.45 (1H, s, OH-7), 10.01 (1H, s, OH-4′), 9.01 (1H, s, OH-3′), 7.50 (1H, d, *J* = 7.7 Hz, H-6′), 7.44 (1H, d, *J* = 1.5 Hz, H-2′), 6.84 (1H, d, *J* = 7.4 Hz, H-5′), 6.60 (1H, s, H-3), 6.21 (1H, s, H-6), 4.62 (1H, d, *J* = 9.0 Hz, H-1″), 3.7–3.6 (2H, m, H-2″, 6″), 3.54 (1H, m, H-6″), 3.49–3.21 (3H, m, H-3″, 4″, 5″); ^13^C-NMR (150 MHz, DMSO-*d_6_*) δ: 180.87 (C-4), 163.94 (C-2), 161.11 (C-7) 160.74 (C-5), 154.74 (C-8a), 149.11 (C-4′), 144.45 (C-3′), 120.78 (C-1′), 118.29 (C-6′), 114.44 (C-5′), 114.14 (C-2′), 104.14 (C-8), 103.45 (C-4a), 101.14 (C-3), 97.74 (C-6), 80.44 (C-5″), 77.64 (C-3″), 72.27 (C-1″), 70.67 (C-2″), 70.64 (C-4″), 60.12 (C-6″).

#### 2.1.8. Gallic Acid

C_7_H_6_O_5_; ESI-QTOF-MS/MS *m/z*: 169.0134 [M − H]^−^, ^1^H-NMR (600 MHz, CD_3_OD-*d_4_*) δ: 7.05 (2H, s, CH); ^13^C-NMR (150 MHz, CD_3_OD-*d_4_*) δ: 170.41 (C=O), 146.36 (C-3, 5), 139.57 (C-4), 121.96 (C-1), 110.32 (C-2, 6).

#### 2.1.9. Caffeic Acid

C_9_H_8_O_4_; ESI-QTOF-MS/MS *m/z*: 179.0342 [M − H]^−^, ^1^H-NMR (600 MHz, DMSO-*d_6_*) δ: 7.40 (2H, d, *J* = 15.8 Hz, H-7), 7.01 (1H, d, *J* = 1.4 Hz, H-2), 6.95 (2H, dd, *J* = 7.6, 2.1 Hz, H-1, 6), 6.74 (2H, d, *J* = 8.3 Hz, H-3, 5), 6.16 (1H, d, *J* = 15.8 Hz, H-8); ^13^C-NMR (150 MHz, DMSO-*d_6_*) δ: 167.95 (C-9), 148.16 (C-4), 145.58 (C-7), 144.63 (C-3), 125.73 (C-1), 121.20 (C-6), 115.77 (C-5), 115.14 (C-8), 114.64 (C-2).

#### 2.1.10. Vitexin-2″-*O*-Rhamnoside

C_27_H_30_O_14_; ESI-QTOF-MS/MS *m/z*: 577.0651 [M − H]^−^, ^1^H-NMR (600 MHz, DMSO-*d_6_*) δ: 8.06 (2H, d, *J* = 9.0 Hz, H-2′, 6′); 6.89 (2H, d, *J* = 9.0 Hz, H-3′, 5′), 6.78 (1H, s, H-3), 6.25 (1H, s, H-6), 4.97 (1H, s, H-1′′′), 4.75 (1H, d, *J* = 9.6 Hz, H-1″), 4.63 (1H, t, *J* = 5.5 Hz, H-1″), 4.41 (2H, d, *J* = 4.1 Hz, H-3′′′), 4.04 (1H, t, *J* = 9.3 Hz, H-1), 3.56 (2H, s, H-2′′′), 3.09–2.91 (9H, m, H-2″~6″, 2, 3, 4,), 2.10 (1H, dd, *J* = 9.6, 6.2 Hz, H-5), 1.89 (1H, s, H-6′′′), 0.46 (3H, d, *J* = 6.2 Hz, H-6″); ^13^C-NMR (150 MHz, DMSO-*d_6_*) δ: 182.09 (C-4), 163.97 (C-2), 162.36 (C-7), 161.19 (C-5), 160.65 (C-4′), 155.81 (C-9), 128.99 (C-6′), 121.58 (C-1′), 115.87 (C-5′), 104.45 (C-10), 104.19 (C-8), 102.42 (C-3), 100.30 (C-1′′′), 98.27 (C-6), 81.80 (C-5″), 79.89 (C-3″), 75.03 (C-2″), 71.65 (C-1″), 71.45 (C-4′′′), 70.64 (C-2′′′), 70.44 (C-3′′′), 70.22 (C-4″), 68.21 (C-5′′′), 61.13 (C-6″), 21.11 (C-1), 17.71 (C-6′′′).

#### 2.1.11. Vitexin-4″-*O*-Glucoside

C_27_H_30_O_15_; ESI-QTOF-MS/MS *m/z*: 593.1464 [M − H]^−^, ^1^H-NMR (600 MHz, CD_3_OD-*d_4_*) δ: 7.98 (2H, d, *J* = 9.0 Hz, H-2′, 6′), 6.94 (1H, d, *J* = 9.0 Hz), 6.60 (2H, s, H-3), 6.24 (2H, d, *J* = 5.5 Hz, H-6), 5.13 (1H, d, *J* = 9.6 Hz, H-1′′′), 5.05 (1H, d, *J* = 9.6 Hz, H-1″), 4.36–3.11 (8H, m, H-2″~6″, 2, 3, 4), 1.95 (1H, s, H-6′′′); ^13^C-NMR (150 MHz, CD_3_OD-*d_4_*) δ: 165.19 (C-2), 161.44 (C-7), 161.30 (C-5), 128.78 (C-3′), 128.35 (C-5′), 122.28 (C-1′), 115.69 (C-4′), 104.77 (C-10), 104.47 (C-8), 102.45 (C-3), 98.12 (C-6), 80.31 (C-5″), 78.88 (C-3″), 75.76 (C-2″), 74.47 (C-2′′′), 72.27 (C-4′′′), 70.71 (C-3′′′), 69.90 (C-4″), 69.72 (C-5′′′), 61.56 (C-6′′′).

### 2.2. Inhibitory Activity of the Extract and Fractions from Benincasae Exocarpium against AGE Formation in BSA-Glucose and BSA-MGO Systems

Advanced glycation end-products (AGEs) produced by hyperglycemia and oxidative stress are major causes of diabetic complications [5,34]. This is because AGEs cause structural and functional changes in proteins [35]. Therefore, inhibition of AGE formation is attracting attention in relation to the prevention of diabetic complications and the development of therapeutic agents [6]. To prove the potential of BE for preventing diabetic complications, we measured the AGE formation inhibitory activity in two systems: bovine serum albumin (BSA)-glucose and BSA-methylglyoxal (MGO). The results are shown in Table 1. In the BSA-glucose system, the extract, EA, and BuOH fractions from BE significantly inhibited AGE formation (IC_50_ values of 684.12 ± 2.82, 628.05 ± 1.73, and 419.31 ± 1.28 µg/mL, respectively). In the BSA-MGO system, the EA and BuOH fractions were the most potent AGE formation inhibitors (IC_50_ values of 214.95 ± 6.38 and 533.43 ± 16.04 µg/mL, respectively). The extract mildly inhibited AGE formation in BSA-MGO (IC_50_ values of 842.38 ± 9.07 µg/mL). On the other hand, the Hx and water fractions showed no or slight inhibition of AGE formation in the two systems.

### 2.3. Inhibitory Activity of the Extract and Fractions from Benincasae Exocarpium against A-Glucosidase

α-Glucosidase is a carbohydrase that catalyzes the liberation of α-glucose from the non-reducing end of the substrate [36]. Its inhibitors delay the absorption of carbohydrates and thus have a lowering effect on postprandial blood glucose and insulin levels [4]. Therefore, they are used in the treatment of type 2 diabetes. We assessed the potential involvement of the inhibition of α-glucosidase by BE, and the results are summarized in Table 1. The EA fraction was found to be most potent in the α-glucosidase inhibitory activity (IC_50_ values of 212.20 ± 4.15 µg/mL). The Hx, BuOH, and water fractions mildly inhibited α-glucosidase (IC_50_ values of 413.38 ± 3.58, 368.72 ± 9.24, 491.18 ± 12.67 µg/mL, respectively. In contrast, the extracts showed no inhibitory activity.

### 2.4. Inhibitory Activity Compounds ***1**–**11*** Isolated from Benincasae Exocarpium against AGE Formation in BSA-Glucose and BSA-MGO Systems

Compounds **1**–**11** were tested for their ability to inhibit AGE formation. The results are shown in Table 2. First, in the BSA-glucose system, five flavonoid compounds (**5**–**7**, **10**, and **11**) showed higher inhibitory activity than AMG as the positive control (IC_50_ values of 20.20 ± 0.10, 19.50 ± 1.47, 9.61 ± 0.27, 29.58 ± 1.02, 14.54 ± 0.39 µM, respectively), as well as compounds **2** and **9** (IC_50_ values of 79.75 ± 1.91 and 54.51 ± 0.30 µM, respectively). Compound **8** had an IC_50_ value of 191.73 ± 2.15 µM, which is similar to AMG. In contrast, compounds **1**, **3**, and **4** showed no activity. In the BSA-MGO system, as in the BSA-glucose system, five flavonoids (**5**–**7**, **10**, and **11**) were more potent inhibitors of AGE formation compared to the other compounds (IC_50_ values of 7.49 ± 0.24, 14.86 ± 3.66, 13.27 ± 0.65, 12.49 ± 0.81 and 11.56 ± 1.10 µM, respectively). Compounds **2** and **9** mildly inhibited AGE formation (IC_50_ values of 53.20 ± 23.24, 43.04 ± 9.41 µM, respectively). In contrast, compounds **1**, **3**, **4**, and **8** showed no or slight inhibitory activity.

### 2.5. Inhibitory Activity of Compounds ***1**–**11*** Isolated from Benincasae Exocarpium against A-Glucosidase

Compound **10** showed the highest inhibitory activity against α-glucosidase (IC_50_ values of 12.82 ± 1.18 µM) compared to acarbose as a positive control (IC_50_ values of 49.46 ± 3.20 µM). Including compound 10, flavonoid compounds (**5**–**7**, and **11**) and compound **2** exhibited IC_50_ values like the positive control (IC_50_ values of 48.19 ± 0.70, 52.28 ± 7.81, 36.14 ± 4.21, 65.38 ± 4.22, and 48.69 ± 5.94 µM, respectively). Compound **8**’s inhibitory activity was also lower than that of the other compounds, but the value was significant (IC_50_ values of 108.65 ± 5.86 µM). In contrast, compounds **1**, **3**, **4**, and **9** showed no, or slight, inhibitory activity. The results are shown in Table 2.

### 2.6. Development and Validation of Simultaneous HPLC-PDA Analysis

For the simultaneous analysis of 11 compounds isolated from BE, the HPLC-PDA method was developed. Among the 11 compounds to be tested, the absorption of each compound was recorded at different wavelengths: *p*-hydroxybenzoic acid (255 nm), protocatechuic acid (260 nm), isovanillin (231 nm), 5-hydroxymethylfurfural (282 nm), isovitexin (334 nm), vitexin (268 nm), orientin (226 nm), gallic acid (213 nm), caffeic acid (325 nm), vitexin-2″-*O*-rhamnoside (268 nm), and vitexin-4″-*O*-glucoside (268 nm). The maximum absorption wavelength of each compound and the peak area at 280 nm were compared and it was confirmed that there was no significant difference between the two cases. Therefore, the detection wavelength was determined to be 280 nm. Subsequently, an analysis method validation was performed. The specificity, linearity, limit of detection (LOD), limit of quantification (LOQ), intra-day and inter-day precision, and accuracy of the analysis method were confirmed. Additionally, the recovery and robustness were also confirmed (see Supplementary Materials, Tables S1 and S2)

#### 2.6.1. Specificity

**Figure 1:**
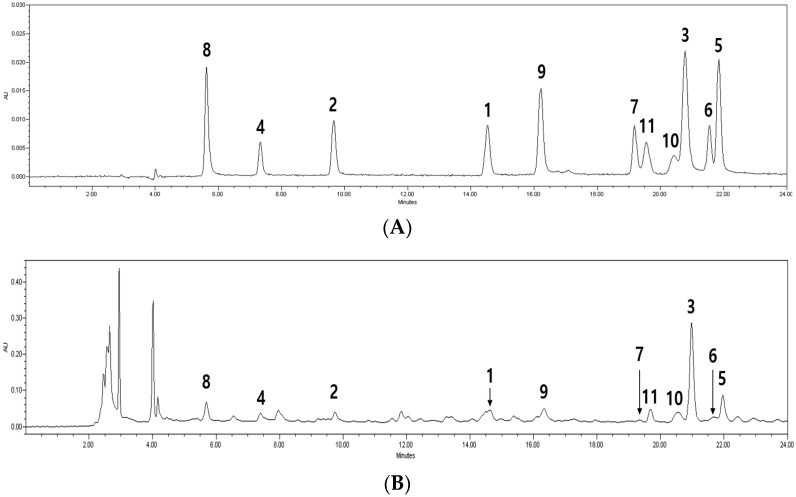
High-performance liquid chromatography (HPLC) chromatograms of standard mixtures (**A**) and Benincasae Exocarpium extracts (**B**). Compound **8** (gallic acid); Compound **4** (5-hydroxymethylfurfural); Compound **2** (protocatechuic acid); Compound **1** (*p*-hydroxybenzoic acid); Compound **9** (caffeic acid); Compound **7** (orientin); Compound **11** (vitexin-4″-*O*-glucoside); Compound **10** (vitexin-2″-*O*-rhamnoside); Compound **3** (Isovanillin); Compound **6** (vitexin); Compound **5** (isovitexin).

By comparing a mixture of 11 compounds with an extract from BE, these were well separated without interference (Figure 1).

#### 2.6.2. Linearity

The linearity of the 11 compounds was measured at five concentrations between 0.5–50 μg/mL. In all calibration curves, the correlation coefficient (r^2^) of all compounds was greater than 0.999, and the results are shown in Table 3.

#### 2.6.3. Limit of Detection (LOD) and Limit of Quantification (LOQ)

The LOD values of 11 compounds ranged from 0.05 to 2.92 μg/mL and LOQ values of all compounds ranged from 0.14 to 8.85 μg/mL (Table 3).

#### 2.6.4. Intra-Day and Inter-Day Precision and Accuracy

Precision and accuracy were determined using 11 compounds mixture solutions. The results are summarized in Table 4. The intra-day and inter-day precisions of all compounds ranged from 0.14% to 5.69% and from 0.12% to 6.19%, respectively. The intra-day and inter-day accuracies of all compounds ranged from 92.1% to 108.6% and from 90% to 102.8%, respectively.

### 2.7. Quantitative HPLC-PDA Analysis of Isolated Compounds

As a result of screening the collected three samples of BE, 11 compounds were identified. Compounds **5** and **10** were identified as the major components of the BE. However, there was a compound with a large difference in the content of the components depending on the type of sample. Therefore, eight (**2**, **5**–**11**) out of the 11 compounds were selected based on the activity assay. The eight bioactive compounds showed strong activity in the anti-glycation (AGE formation inhibitory) and anti-diabetic (α-glucosidase inhibitory) assays. HPLC-PDA analysis of the BE extracts was performed to quantitatively evaluate the bioactive compounds (Table 5). To optimize the extraction efficiency of these compounds, BE samples underwent extraction by altering the extraction solvent, (methanol [MeOH] and ethanol [EtOH]), the solvent ratios (30, 50, 70, and 100%), and the time (30, 60, 90, and 120 min). The sum of the peak areas of eight compounds was compared. Among the various extraction conditions, extraction done with 70% EtOH and during 60 min produced samples that contained the most eight marker compounds.

The compounds of BE showed high contents in the following order: Compound **5** (isovitexin, 4.798 ± 0.039 mg/g) > Compound **10** (vitexin-2″-*O*-rhamnoside, 3.780 ± 0.009 mg/g) > Compound **2** (protocatechuic acid, 1.260 ± 0.004 mg/g) > Compound **7** (orientin, 1.115 ± 0.007 mg/g) > Compound **11** (vitexin-4″-*O*-glucoside, 0.909 ± 0.027 mg/g) > Compound **9** (caffeic acid, 0.790 ± 0.025 mg/g) > Compound **6** (vitexin, 0.768 ± 0.008 mg/g) > Compound **8** (gallic acid, 0.572 ± 0.003 mg/g). Therefore, we proposed that the content criteria of BE be 0.113% for compound **2**, 0.432% for compound **5**, 0.069% for compound **6**, 0.101% for Compound **7**, 0.051% for Compound **8**, 0.071% for Compound **9**, 0.340% for Compound **10**, and 0.081% for Compound **11**.

## 3. Materials and Methods

### 3.1. Plant Material

Benincasae Exocarpium was purchased from the Kyung-dong market in Seoul, Korea. In addition, *B. cerifera* samples were collected from Cheonan, Korea. They were authenticated by Professor, Wan Kyunn Whang (College of Pharmacy, Chung-Ang University, Seoul, Korea).

### 3.2. Instruments and Reagents

Methanol (MeOH), ethanol (EtOH), *n*-hexane (Hx), ethyl acetate (EtOAc), *n*-butanol (BuOH) and distilled water were used for extraction, fractionation, and open column chromatography. An octadecylsilane (ODS) gel (75 µm) (YMC Co., Ltd., Kyoto, Japan), Sephadex LH-20 (25–100 µm) (Pharmacia, Milford, MA, USA) and MCI CHP 20P gel (Supelco, St. Louis, MO, USA) were used for open column chromatography.

^1^H- and ^13^C-nuclear magnetic resonance (NMR) spectra were recorded at 600 and 150 MHz, respectively, using a JEOL spectrometer (JEOL, Akishima, Tokyo, Japan). Chemical shifts are presented as parts per million (ppm) on the δ scale, and the coupling constants (*J*) are shown in Hertz. Chloroform-*d* (CDCl_3_-*d*), dimethyl sulfoxide-*d_6_* (DMSO-*d_6_*), and methanol-*d_4_* (CD_3_OD-*d_4_*) were purchased from Sigma-Aldrich Co. (Sigma-Aldrich Co., St. Louis, MO, USA) as solvents for NMR analysis.

Electrospray ionization quadrupole time-of-flight mass spectrometry (ESI-QTOF-MS/MS) was performed using Bruker compact data analysis software version 4.3, a Bruker compact (Bruker, Billerica, MA, USA). High-performance liquid chromatography-photodiode array detector (HPLC-PDA) was analyzed using Empower Pro 2.0, a Waters 2695 system pump equipped with a Waters 2489 photodiode array detector (Waters, Milford, MA, USA). The HPLC column was a Fortis C_18_ column (4.6 × 250 mm, 5 μm, Waters, Milford, MA, USA). HPLC-grade solvents (e.g., acetonitrile, distilled water, and acetic acid) were obtained from Thermo Fisher Scientific (Waltham, MA, USA).

Among the anti-diabetes activity assays, the Infinite^®^ F200 PRO spectrophotometer (TECAN, Männedorf, Zürich, Switzerland) was used for the AGE formation inhibitory activity assay in the BSA-glucose and BSA-MGO systems. In addition, the α-glucosidase inhibitory activity assay was performed using Biotek EPOCH2 (Biotek, Winooski, VT, USA). Regarding the activity assays, sodium azide, sodium phosphate dibasic, sodium phosphate monobasic, aminoguanidine hydrochloride (AMG), bovine Serum Albumin (BSA), DMSO, D-(−)-fructose, D-(+)-glucose, methylglyoxal (MGO), α-glucosidase from *Saccharomyces cerevisiae* (Saccharomycetaceae), 4-nitrophenyl α-D-glucopyranoside (p-NPG) and acarbose were purchased from Sigma-Aldrich, Co. (Sigma-Aldrich Co., St. Louis, MO, USA).

### 3.3. Extraction, Fractionation, and Isolation of Benincasae Exocarpium

The BE (4.0 kg) was dried, powdered, and then extracted in methanol (20 L × 3) at room temperature. The filtrate was concentrated to dryness (406.03 g) *in vacuo*, suspended in water (H_2_O), and then partitioned using Hx, EtOAc, and BuOH. The results yielded Hx (100.46 g), EtOAc (21.15 g), BuOH (35.74 g), and water (154.22 g) fractions. Among these four fractions, the EtOAc and BuOH fractions were the most potent in the three anti-diabetes activity assays. In addition, in the fraction analysis using HPLC-PDA, various polar components were confirmed in the two fractions. Therefore, repeated open column chromatography of both active fractions was performed.

The EtOAc fraction (21.15 g) was subjected to silica gel open column chromatography with CHCl_3_:MeOH:water (70:30:4→50:50:4), and four sub-fractions were obtained. Sub-fraction EtOAc 1 was isolated using MCI gel column chromatography with 50% MeOH to obtain sub-fraction EtOAc 1-3. Sub-fraction EtOAc 1-3 was separated using a Sephadex LH-20 column and eluted with 20% MeOH to obtain sub-fraction EtOAc 1-3-2. Sub-fraction EtOAc 1-3-2 was subjected to MCI gel column chromatography using 30% EtOH to obtain compound **1**. To increase the purity of compound **1**, Sephadex LH-20 column with EtOH 50% was performed. Sub-fraction EtOAc 2 was applied to a Sephadex LH-20 open column and eluted with 20% to 70% MeOH to obtain two sub-fractions (sub-fraction EtOAc2-1 to EtOAc2-2). Sub-fraction EtOAc 2-1 was applied to Sephadex LH-20 and eluted with 30% EtOH to obtain compound **2**. Sub-fraction EtOAc 2-2 was subjected to ODS column chromatography with 10% EtOH to obtain compound **3**. Sub-fraction EtOAc 3 was subjected to ODS column chromatography with 30% MeOH to obtain sub-fraction EtOAc 3-2. Sub-fraction EtOAc 3-2 was applied to Sephadex LH-20 column with 50% MeOH to obtain compound **4**. Sub-fraction EtOAc 4 was applied to a Sephadex LH-20 column with 50% MeOH to obtain two sub-fractions (sub-fraction EtOAc 4-2 and EtOAc 4-3). Sub-fraction EtOAc 4-2 was subjected to MCI gel column chromatography with 30% EtOH to obtain compound **5**. Sub-fraction EtOAc 4-3 was subjected to MCI gel column chromatography with 10% MeOH to sub-fraction obtain EtOAc 4-3-1 and EtOAc 4-3-2. Sub-fraction EtOAc 4-3-1 was applied to a Sephadex LH-20 column with 30% EtOH to obtain compound **6**. Sub-fraction EtOAc 4-3-2 was subjected to ODS column chromatography with 10% MeOH to obtain compound **7**.

The BuOH fraction (35.7 g) was subjected to silica gel open column chromatography with CHCl_3_:MeOH (90:10→30:60). Two sub-fractions were obtained (sub-fraction BuOH 1, sub-fraction BuOH 2). Sub-fraction BuOH 1 was subjected to MCI gel column chromatography with 40% MeOH and two sub-fractions (sub-fraction BuOH 1-3 and BuOH 1-5) were obtained. The BuOH 1-3 sub-fractions was applied to a Sephadex LH-20 column with 10% EtOH to yield compound **8**. In addition, sub-fraction BuOH 1-5 was subjected to ODS column chromatography with 5% MeOH to obtain compound **9**. Sub-fraction BuOH 2 was applied to a Sephadex LH-20 column with 20% MeOH to obtain sub-fractions BuOH 2-1 and BuOH 2-3. Sub-fraction BuOH 2-1 was subjected to ODS column chromatography with 20% MeOH and yielded the sub-fraction BuOH 2-1-3. Sub-fraction BuOH 2-1-3 was applied to a Sephadex LH-20 column with 10% EtOH to obtain compound **10**. Finally, sub-fraction BuOH 2-3 was applied to a Sephadex LH-20 column with 50% MeOH to obtain sub-fraction BuOH 2-3-1. Sub-fraction BuOH 2-3-1 was subjected an MCI gel column with 10% MeOH to obtain sub-fraction BuOH 2-3-2. Sub-fraction BuOH 2-3-2-1 was applied to Sephadex LH-20 with 10% EtOH to obtain compound **11**.

### 3.4. Identification Method of Isolated Compounds from Benincasae Exocarpium

#### 3.4.1. H and ^13^C NMR Spectroscopy Analysis

Each isolated compound (**1**–**11**) was weighed (8 mg) and dissolved in 800 µL of each solvent (CDCl_3_-*d*, CD_3_OD-*d_4_*, and DMSO-*d_6_*). The optimal ^1^H and ^13^C NMR spectroscopic analytical conditions were as follows: ^1^H NMR: scans-30, relaxation delay-10, and gain-optimal selection; ^13^C NMR: scans-1800, relaxation delay-30, gain-optimal selection.

#### 3.4.2. ESI-QTOF-MS/MS Analysis

The molecular weights of the isolated compounds **1**–**11** were determined using ESI-QTOF-MS/MS. Isolated compounds (1 mg) were dissolved in 10 mL of MeOH and filtered using a 0.45 µm syringe filter. The prepared compounds were directly injected into a Bruker Compact system.

The mobile phase was run on a gradient schedule with solvent A (water, 0.1% formic acid, *v*/*v*) and solvent B (acetonitrile, 0.1% formic acid, *v*/*v*). Solvent B was increased from 10% to 50% for 5 min and then from 50% to 100% for 10 min, maintained for 3 min, decreased from 100% to 10%, and maintained for 5 min. The flow rate of the mobile phase was 0.2 mL/min, and the injection volume was 30 µL. The optimal ESI-QTOF-MS/MS analysis conditions were as follows: source type electrospray ionization, ion polarity-negative, scan-50–800 *m/z*, set capillary-4500 V, set endplate offset-500 V, set nebulizer-1.2 bar, set dry heater-200 °C, and set dry gas-10.0 L/min.

### 3.5. HPLC-PDA Analysis

To analyze the isolated compounds (**1**–**11**), a Fortis C_18_ Column (4.6 × 250 mm, 5 μm, Waters, Milford, MA, USA) was used. Mobile phases A and B consisted of water containing 1% acetic acid and acetonitrile, respectively, and were run on the following gradient schedule. Mobile phase A was maintained for 24 min and increased from 10% to 27%. The flow rate of the mobile phase was 1.0 mL/min, and the injection volume was 10 µL. Column temperature was maintained at 25 °C. Detection was performed at a UV absorbance wavelength of 280 nm. This analysis method is validated by various evaluation sections including specificity, linearity, LOD and LOQ, and intra- and inter-day precision and accuracy [37]. The samples were prepared as follows. Powdered BE samples (1 g) were dissolved in different solvents (MeOH: 30%, 50%, 70%, and 100%; EtOH: 30%, 50%, 70%, and 100%) and sonicated for different times (30, 60, 90, and 120 min). In addition, each sample was filtered using filter paper, and the solvent was removed in vacuo. The dried samples were dissolved in 1 mL of MeOH, filtered through a 0.45 μm syringe filter, and used as a sample solution. 1 mg of isolated compounds **1**–**11** were dissolved in 1 mL of MeOH and filtered through a 0.45 μm syringe filter. The compounds (**1**–**11**) were mixed in the same ratio to make a standard solution. They were diluted to concentrations between 2.5–50 ug/mL and used for HPLC-PDA analysis to validate the analysis conditions.

### 3.6. Bioactivity Assays

#### 3.6.1. Inhibitory Activity against AGE Formation in BSA-Glucose System

The AGE formation inhibitory assay of BSA-glucose was conducted using a spectrophotometric method developed in a previous study [38]. All test samples were dissolved in 10% DMSO at five different concentrations (1000–10,000 μg/mL for the extract and fractions and 100–10,000 μM for the compounds). The assay mixtures contained the following constituents: 135 µL of 50 mM phosphate buffer (pH 7.4) containing 0.02% sodium azide and BSA (10 mg/mL), 135 µL of 0.4 M D-fructose and D-glucose, and 30 µL of sample. The mixture was incubated at 60 °C for 2 days. After incubation, the fluorescence was measured at an excitation wavelength of 350 nm and emission wavelength of 450 nm in a 96-black well plate. AMG was used as a positive control. The inhibitory activity on AGE formation of BSA-glucose was calculated using the following formula: {(Ac − As)/Ac} × 100, where Ac is the fluorescence of the control and As is the fluorescence of the sample. Controls contained the same reaction mixture, except that phosphate buffer was added instead of the sample. The half-maximal inhibitory concentration (IC_50_) values of triplicate measurements of the samples and AMG were calculated and expressed as the mean ± SD.

#### 3.6.2. Inhibitory Activity against AGE Formation in BSA-MGO System

The AGE formation inhibitory assay of BSA-MGO was conducted using spectrophotometry according to a previously described method with modification [38,39]. All samples were dissolved in 10% DMSO. The assay mixtures contained 50 µL of 50 mM phosphate buffer (pH 7.4) containing 0.02% sodium azide and BSA (10 mg/mL), 50 µL of 7 mM MGO, and 50 µL of sample. After incubation at 60 °C for 2 days, fluorescence was measured on excitation and emission wavelengths of 340 and 420 nm, respectively, in a 96-black well plate. AMG was used as a positive control. The inhibitory activity on AGE formation by BSA-MGO was calculated using the same equation applied in the BSA-glucose assay.

#### 3.6.3. Inhibitory Activity against α-Glucosidase

α-Glucosidase inhibitory activity was measured spectrophotometrically according to the previously described method [40]. All samples were dissolved in 10% DMSO. This assay consisted of a 50 μL sample solution, 50 μL of 50 mM potassium phosphate buffer (pH 6.8), and 50 μL of 0.5 U/mL α-glucosidase was preincubated for 10 min at 37 °C. After incubation, 50 μL of p-NPG was added to the mixed assay mixtures. The reaction was performed in a 96-well plate, and the activity was measured using a spectrophotometer at 405 nm with acarbose as a positive control. The inhibitory activity was calculated using the equation applied in the BSA-glucose assay, but Ac and As are the absorbance values of the control and sample, respectively.

### 3.7. Statistical Analysis

Statistical significance was measured using one-way analysis of variance (ANOVA) and multiple comparisons with *p* < 0.05, *p* < 0.01, and *p* < 0.001 indicated statistical significance.

## 4. Conclusions

In this study, we confirmed the inhibitory activity of extracts and fractions of BE against AGE formation and α-glucosidase. Since EA and BuOH fractions exhibited the strongest inhibitory activities, we isolated 11 compounds (seven from EA fractions and four from BuOH fractions) in accordance with bioassay-guided isolation. They were identified by ^1^H, ^13^C-NMR, and ESI-QTOF-MS/MS. AGE formation and α-glucosidase inhibitory activity were confirmed for compounds **1**–**11**. Among the isolated compounds, five flavonoids (**5**, **6**, **7**, **10**, and **11**) showed stronger inhibitory activity against AGE formation in BSA-glucose and BSA-MGO systems than in positive controls. In addition, these compounds showed similar or high α-glucosidase inhibitory activity to the positive control group. Compound **2** showed high inhibitory activity against alpha-glucosidase and significant inhibition of AGE formation in BSA-MGO and BSA-glucose systems. Compounds **8** and **9** also showed significant inhibition of AGE formation in the BSA-glucose system. Compounds **1**, **3**, and **4** showed no or slight inhibitory activity. HPLC-PDA simultaneous analysis was developed for separated compounds **1**–**11**, and validation was performed. Using the developed analysis method, the content of eight bioactive compounds (**2** and **5**) among compounds **1**–**11** was confirmed. The optimized extraction conditions for the eight compounds were 70% EtOH and 60 min of extraction. In addition, by presenting content standards for eight compounds, standards for quality management were prepared.

In summary, a systematic experiment (activity assays, compound isolation, and development of a multi-compound simultaneous content analysis method) was conducted to confirm that BE has an anti-diabetic effect. We confirmed anti-diabetic activity of the BE extract and its fractions. The various compounds were separated by open column chromatography, and anti-diabetic activity assays of the active ingredients were performed. In addition, the content standard of the BE was set through a multi-component simultaneous content analysis method using HPLC-PDA.

Based on these results, the possibility of BE as a functional material for the treatment of type 2 diabetes and diabetes complications was confirmed. Additional studies, including in vivo studies of BE, need to be conducted to evaluate whether efficacy is sufficient for clinical application and the drug effects of isolated compounds. Furthermore, compounds isolated from BE may be valuable functional agents against other diseases.

## Figures and Tables

**Table 1:** IC_50_ (inhibitory activity) of the Benicasae Exocarpium (BE) extract and fractions against advanced glycation end-products (AGEs) formation in bovine serum albumin (BSA)-glucose and BSA-methylglyoxal (MGO) systems and α-glucosidase.

Sample	IC_50_^a^ (μg/mL)		
	BSA-Glucose	BSA-MGO	α-Glucosidase
Extract	684.12 ± 2.82 ***	842.38 ± 9.07 ***	>500
Hx fraction	ND ^d^	ND ^d^	413.38 ± 3.58 **
EA fraction	628.05 ± 1.73 **	214.95 ± 6.38 ***	212.20 ± 4.15 ***
BuOH fraction	419.31± 1.28 ***	533.43 ± 16.04 **	368.72 ± 9.24 **
Water fraction.	1875.93 ± 3.02 ***	1102.80 ± 4.36 *	491.18 ± 12.67 ***
AMG ^b^	217.93 ± 1.54 ***	167.32 ± 8.40 ***	-
Acarbose ^c^	-	-	44.98 ± 1.97 ***

Data are expressed as the mean ± SD (*n* = 3); ^a^ IC_50_ was calculated from the least-squares regression line of the logarithmic concentrations plotted against the residual activity; ^b^ aminoguanidine hydrochloride (AMG) was used as a positive control of AGE formation inhibitory activities; ^c^ Acarbose was used as a positive control for α-glucosidase inhibitory activity; ^d^ ND: not detected; * indicates a significant difference from control; * *p* < 0.05, ** *p* < 0.005, *** *p* < 0.001; -: not measured.

**Table 2:** IC_50_ (inhibitory activity) of compounds **1**–**11** against AGE formation in BSA-glucose and BSA-MGO systems and α-glucosidase.

Compound	IC_50_^a^ (μM)		
	BSA-Glucose	BSA-MGO	α-Glucosidase
**1**	ND ^d^	ND ^d^	>1000
**2**	79.75 ± 1.91 ***	53.20 ± 23.24 ***	48.69 ± 5.94 ***
**3**	ND ^d^	> 500	310.45 ± 13.87 **
**4**	ND ^d^	380.78 ± 14.72 ***	ND ^d^
**5**	20.20 ± 0.10 ***	7.49 ± 0.24 **	48.19 ± 0.70 ***
**6**	19.50 ± 1.47 ***	14.86 ± 3.66 **	52.28 ± 7.81 ***
**7**	9.61 ± 0.27 ***	13.27 ± 0.65 ***	36.14 ± 4.21 **
**8**	191.73 ± 2.15 ***	>500	108.65 ± 5.86 ***
**9**	54.51 ± 0.30 ***	43.04 ± 9.41 **	ND ^d^
**10**	29.58 ± 1.02 ***	12.49 ± 0.81 ***	12.82 ± 1.18 **
**11**	14.54 ± 0.39 **	11.56 ± 1.10 ***	65.38 ± 4.22 *
AMG ^b^	178.86 ± 1.15 **	25.44 ± 0.68 ***	-
Acarbose ^c^	-	-	49.46 ± 3.20 **

Data are expressed as the mean ± SD (*n* = 3); ^a^ IC_50_ was calculated from the least-squares regression line of the logarithmic concentrations plotted against the residual activity; ^b^ AMG was used as a positive control for AGE formation inhibitory activities; ^c^ Acarbose was used as a positive control for α-glucosidase inhibitory activity; ^d^ ND: not detected; * indicates a significant difference from control; * *p* < 0.05, ** *p* < 0.005, *** *p* < 0.001; -: not measured.

**Table 3:** Calibration curves and Linear range of compounds **1**–**11**.

Compound	Regression Equation ^a^	Correlation Coefficient (r^2^) ^b^	Linear Range (μg/mL)	LOD (μg/mL)	LOQ (μg/mL)
**1**	y = 1086.7 x + 155.51	0.9994	5*–*50	0.13	0.38
**2**	y = 1068 x − 1122.5	0.9996	2.5*–*50	0.35	1.06
**3**	y = 3276.6 x − 1292.1	0.9990	2.5*–*50	0.57	1.73
**4**	y = 594.27 x − 0.3417	0.9996	2.5*–*50	0.06	0.19
**5**	y = 830.1 x + 603.75	0.9991	2.5*–*50	0.76	2.31
**6**	y = 2127.5 x − 666.01	0.9993	2.5*–*50	0.68	2.08
**7**	y = 909.98 x + 208.53	0.9996	5–50	0.24	0.71
**8**	y = 1725.5 x − 380.22	0.9996	2.5*–*50	0.75	2.26
**9**	y = 2154.2 x − 1761	0.9990	2.5*–*50	0.71	2.16
**10**	y = 540.76 x + 559.67	0.9997	5*–*50	1.50	4.56
**11**	y = 1046.6 x − 2386.6	0.9997	5*–*50	0.39	1.17

Each value was presented by calculating the mean of triplication; ^a^ Y = peak area, x = concentration of standard (μg/mL); ^b^ r^2^ = correlation coefficient for five final concentrations in the calibration curve; LOD, limit of detection; LOQ, limit of quantification.

**Table 4:** Intra-day and inter-day precision and accuracy of compounds **1**–**11**.

Compound	Con. (μg/mL)	Precision (C.V.%)		Accuracy (%)	
		Intra-Day	Inter-Day	Intra-Day	Inter-Day
**1**	100	1.29	2.97	99.6	95.8
	60	3.91	3.25	96.8	96.6
	40	0.72	2.11	100.4	99.2
**2**	100	0.14	3.49	105.9	97.3
	60	0.85	1.93	97.4	95.2
	40	1.28	3.35	97.5	101.7
**3**	100	1.85	0.56	102.8	90.0
	60	0.86	3.51	94.8	95.7
	40	3.89	4.14	94.5	95.1
**4**	100	3.19	3.71	101.7	99.3
	60	5.69	1.93	98.6	95.7
	40	4.05	3.68	102.0	102.8
**5**	100	1.37	0.98	98.7	99.5
	60	2.15	1.24	96.4	96.7
	40	1.63	1.07	97.9	95.8
**6**	100	4.89	0.62	96.9	91.9
	60	0.82	6.19	92.1	90.6
	40	2.57	4.47	94.3	99.0
**7**	100	0.99	3.41	96.0	91.7
	60	2.32	0.52	94.1	92.0
	40	2.72	2.86	96.1	96.7
**8**	100	0.85	4.34	108.5	99.2
	60	0.71	1.81	98.1	95.9
	40	2.22	1.93	95.5	98.6
**9**	100	0.21	1.93	108.6	95.0
	60	0.51	4.09	97.1	95.8
	40	0.24	1.44	96.4	98.2
**10**	100	3.27	5.31	100.2	96.5
	60	1.54	1.61	92.8	93.8
	40	3.75	6.14	98.2	100.1
**11**	100	3.45	0.12	98.3	94.5
	60	2.07	3.16	101.2	99.7
	40	2.51	2.04	102.6	97.0

Each value was presented by calculation mean of triplication.

**Table 5:** Content of compounds **2** and **5**–**11** in the Benincasae Exocarpium.

**Sample**	**Contents (mg/g)**			
	**Compound 2**	**Compound 5**	**Compound 6**	**Compound 7**
BE 1	0.910 ± 0.001	5.614 ± 0.070	0.697 ± 0.012	1.236 ± 0.006
BE 2	1.410 ± 0.005	5.440 ± 0.024	0.860 ± 0.008	1.118 ± 0.004
BE 3	1.460 ± 0.005	3.340 ± 0.024	0.747 ± 0.004	0.993± 0.013
Mean	1.260 ± 0.004	4.798 ± 0.039	0.768 ± 0.008	1.115 ± 0.007
**Sample**	**Contents (mg/g)**			
	**Compound 8**	**Compound 9**	**Compound 10**	**Compound 11**
BE 1	0.695 ± 0.004	0.649 ± 0.008	3.243 ± 0.005	0.239 ± 0.006
BE 2	0.699 ± 0.004	0.713 ± 0.011	4.845 ± 0.009	1.871 ± 0.065
BE 3	0.322 ± 0.001	1.010 ± 0.056	3.254 ± 0.014	0.619 ± 0.010
Mean	0.572 ± 0.003	0.790 ± 0.025	3.780 ± 0.009	0.909 ± 0.027

Each value was presented by calculation mean of triplication.

## Supplementary Materials

The following are available online. Figures S1–S33: NMR and MS data of compound **1**–**11**; Table S1: Recovery of compounds (**1**–**11**) for three different spiked concentrations; Table S2: Robustness of compounds (**1**–**11**) under various analytical conditions.
