# Supplementary material for: Isolation of Anti-Diabetic Active Compounds from Benincasae Exocarpium and Development of Simultaneous Analysis by HPLC-PDA
Source: Molecules. 2021 Dec 21;27(1):9. doi: 10.3390/molecules27010009 (PMC8746645; doi:10.3390/molecules27010009)
Supplement: Supplementary file 1 [file molecules-27-00009-s001.zip › molecules-1506758-supplementary.pdf]

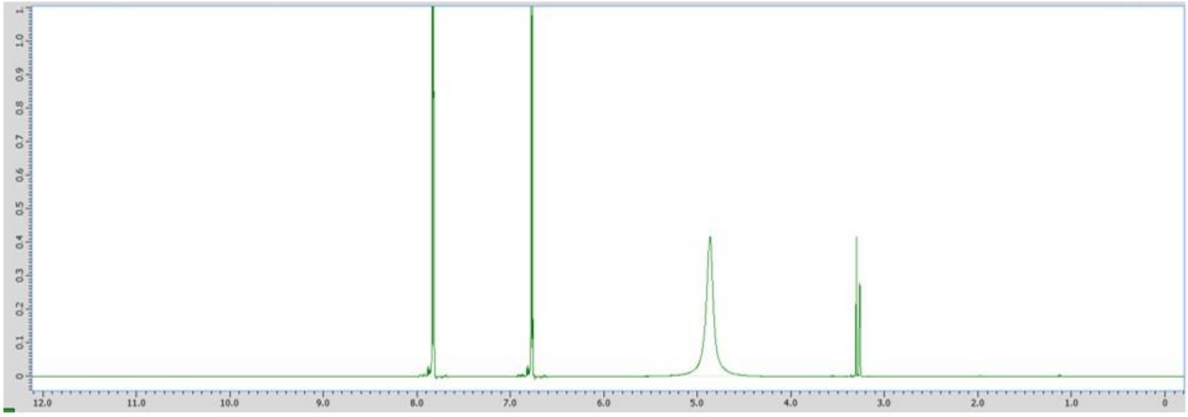

Figure S1. ^{1}H-NMR (600MHz, CD_3OD-d_4) spectrum of compound **1**

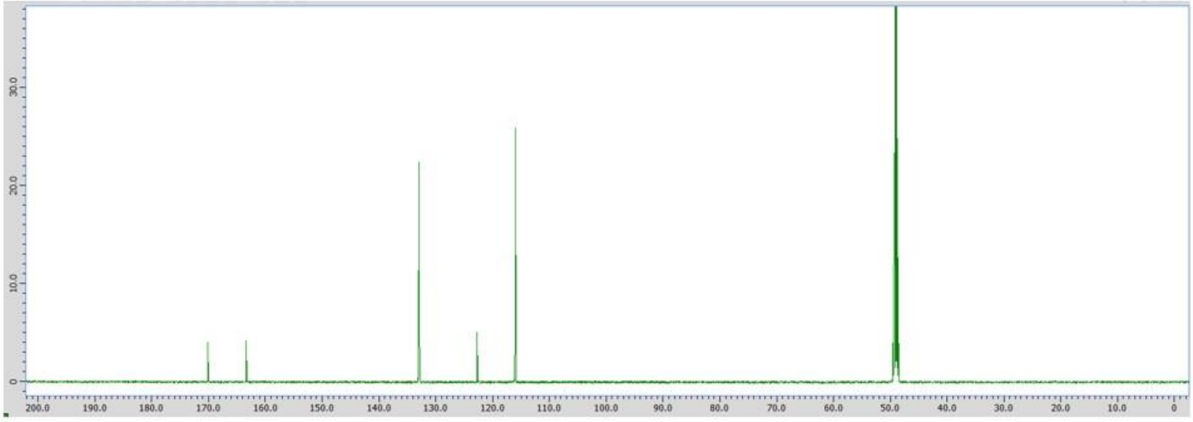

Figure S2. ^{13}C-NMR (150MHz, CD_3OD-d_4) spectrum of compound **1**

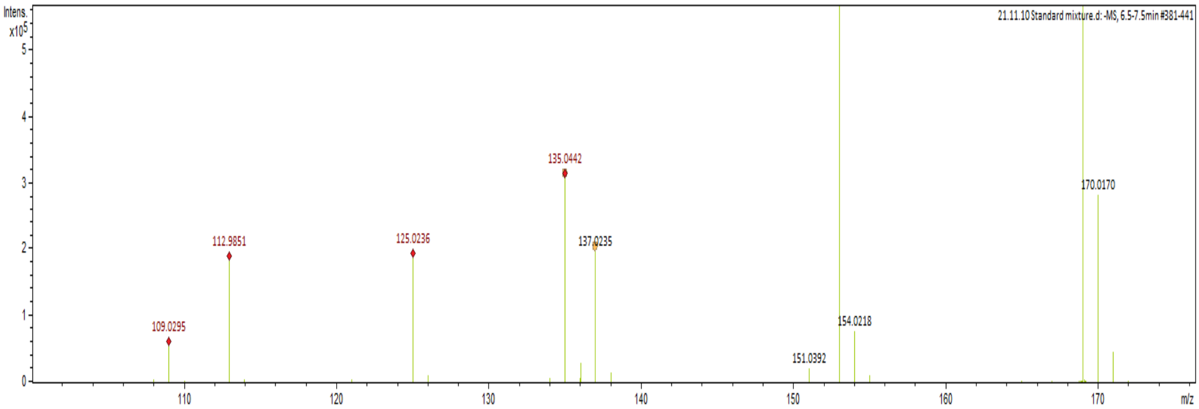

Figure S3. ESI/LTQ-Orbitrap-HRMS spectrum of compound **1** (negative mode)

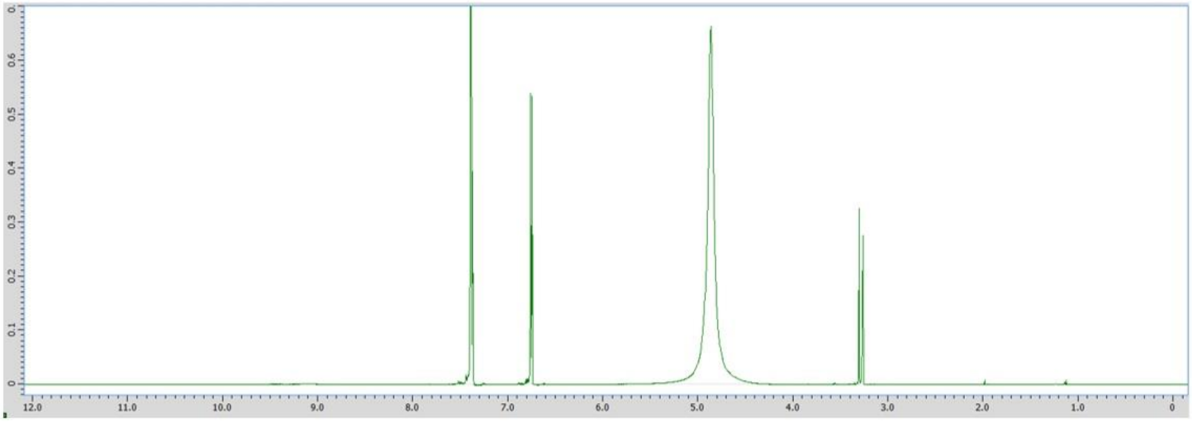

Figure S4. ^{1}H-NMR (600MHz, CD_3OD-*d*$_4$) spectrum of compound **2**

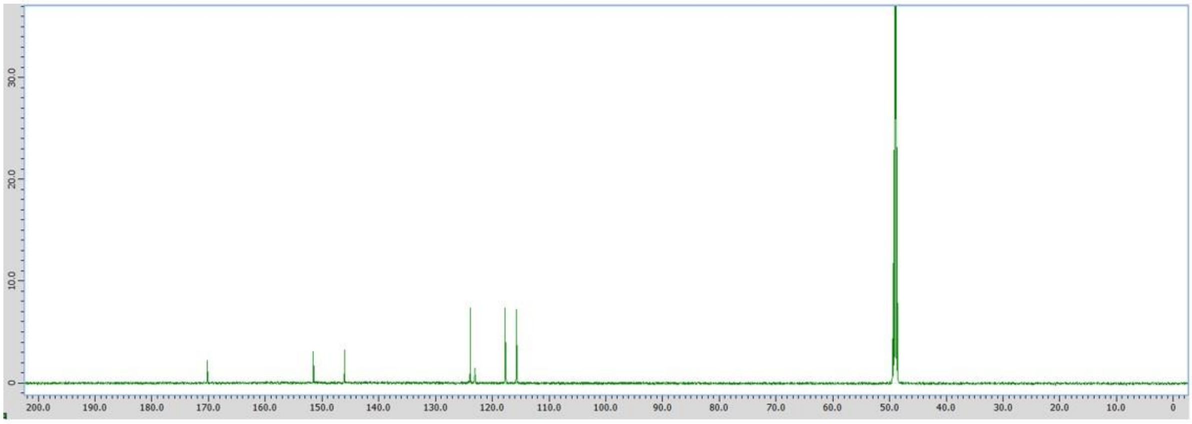

Figure S5. ^{13}C-NMR (150MHz, CD_3OD-*d*$_4$) spectrum of compound **2**

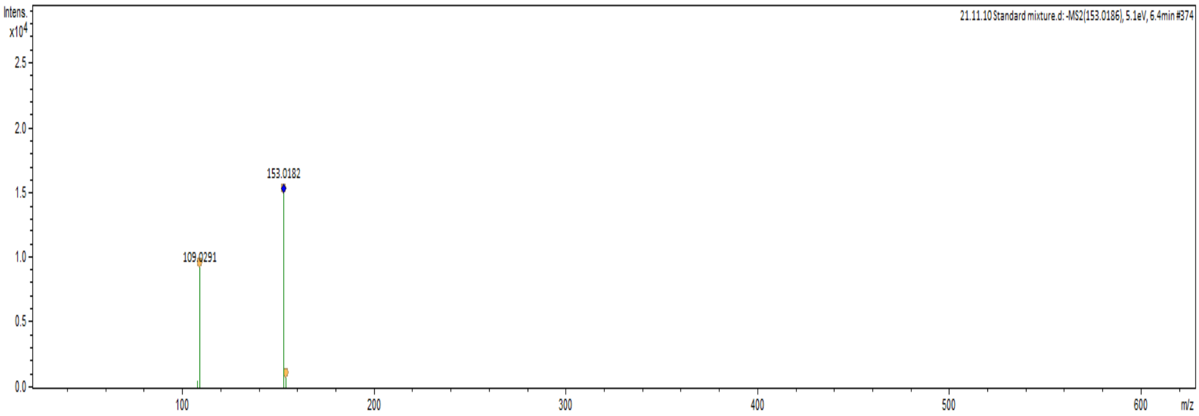

Figure S6. ESI/LTQ-Orbitrap-HRMS spectrum of compound **2** (negative mode)

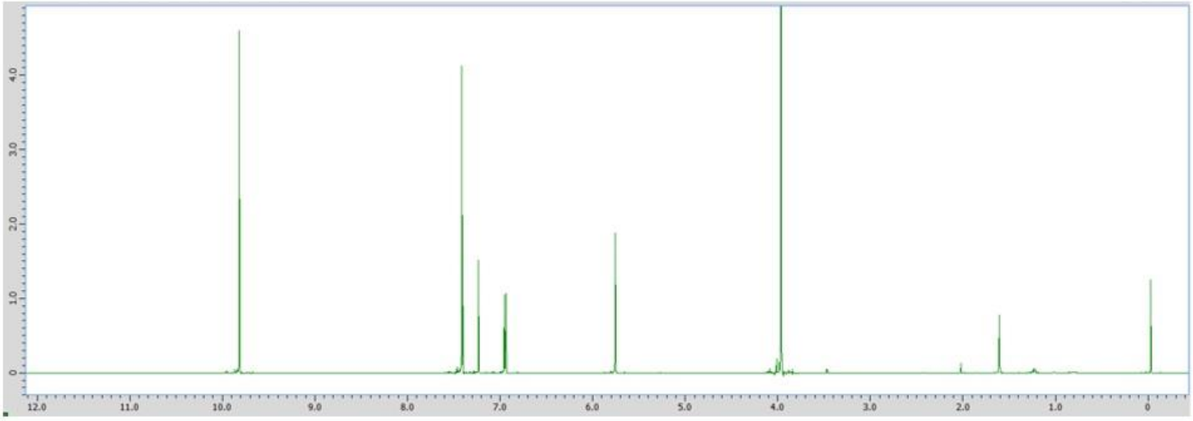

Figure S7. ^{1}H-NMR (600MHz, $CDCl_3$-*d*) spectrum of compound **3**

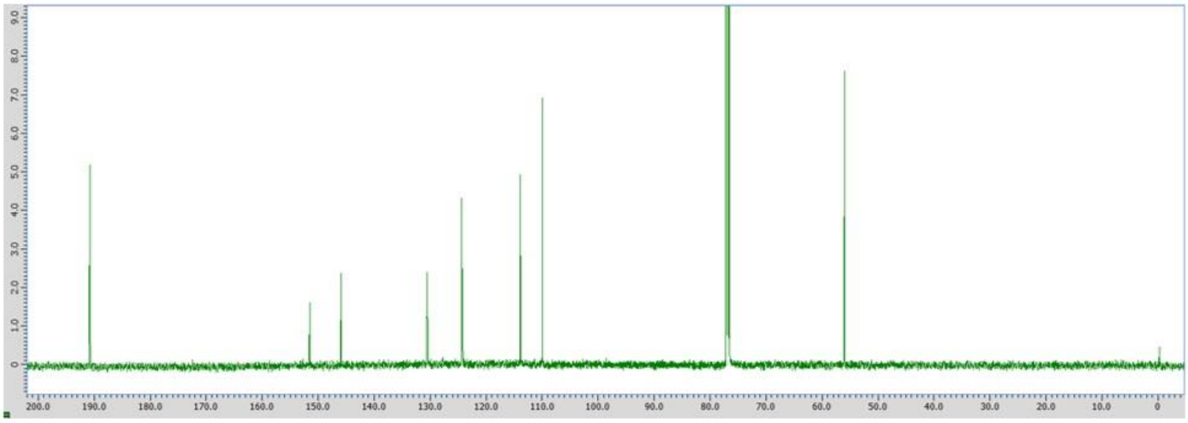

Figure S8. ^{13}C-NMR (150MHz, $CDCl_3$-*d*) spectrum of compound **3**

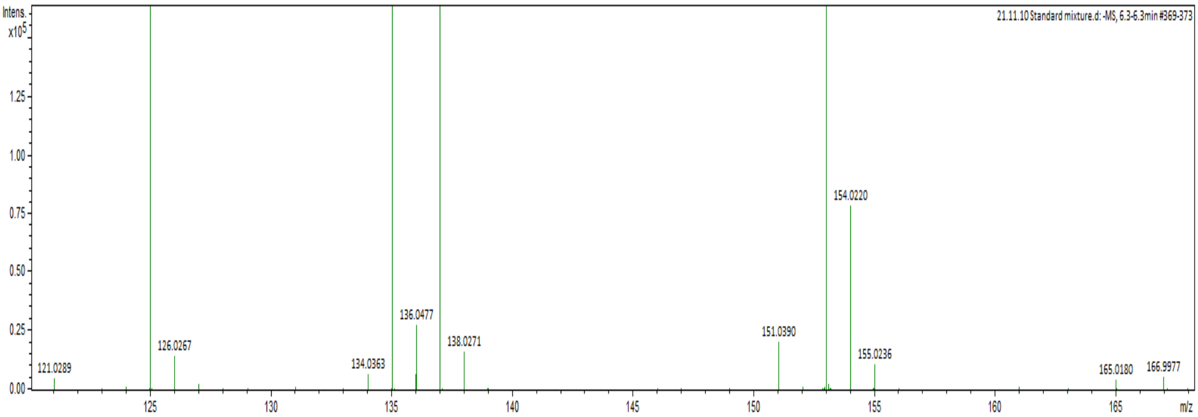

Figure S9. ESI/LTQ-Orbitrap-HRMS spectrum of compound **3** (negative mode)

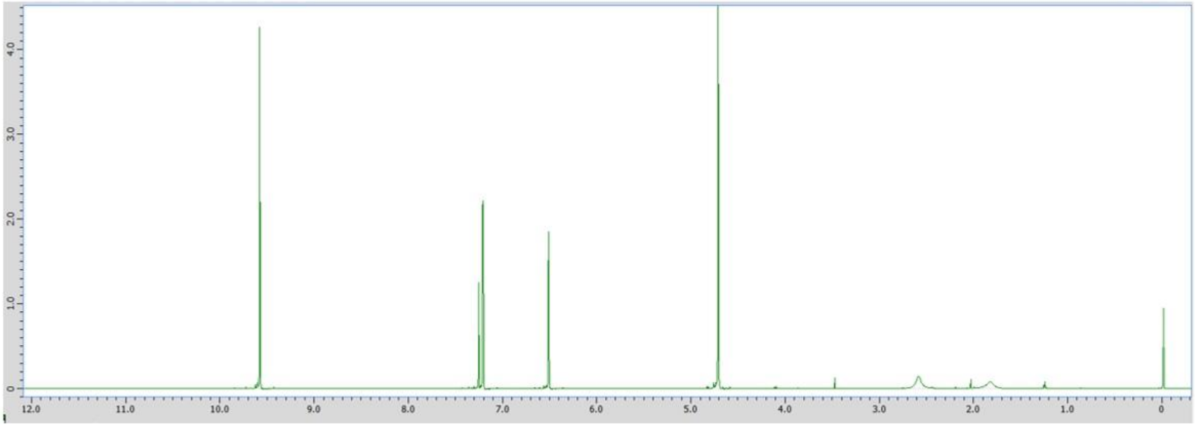

Figure S10. ^{1}H-NMR (600MHz, $CDCl_3$-*d*) spectrum of compound **4**

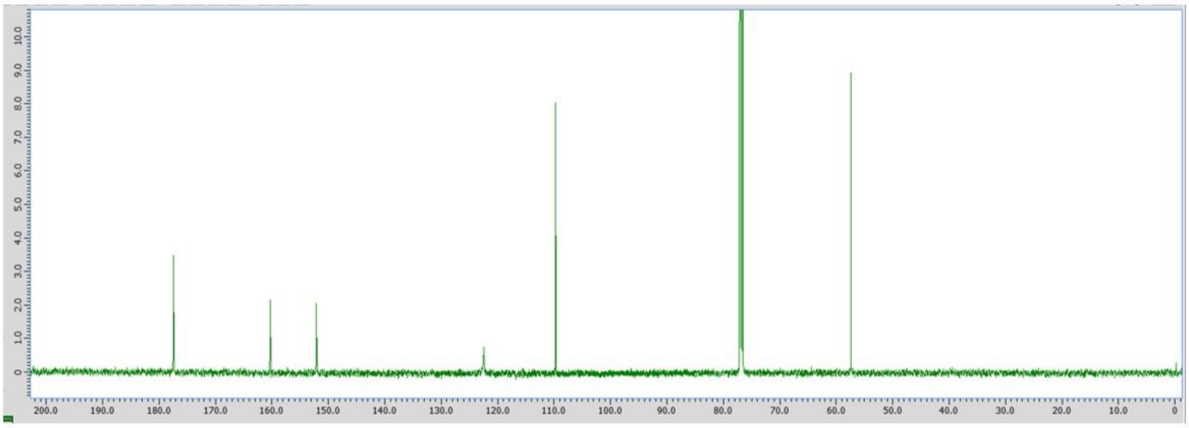

Figure S11. ^{13}C-NMR (150MHz, $CDCl_3$-*d*) spectrum of compound **4**

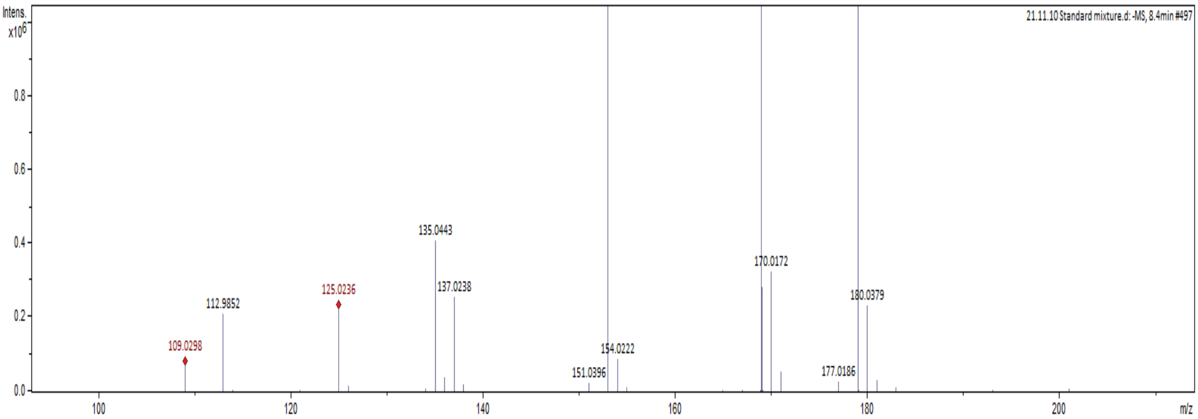

Figure S12. ESI/LTQ-Orbitrap-HRMS spectrum of compound **4** (negative mode)

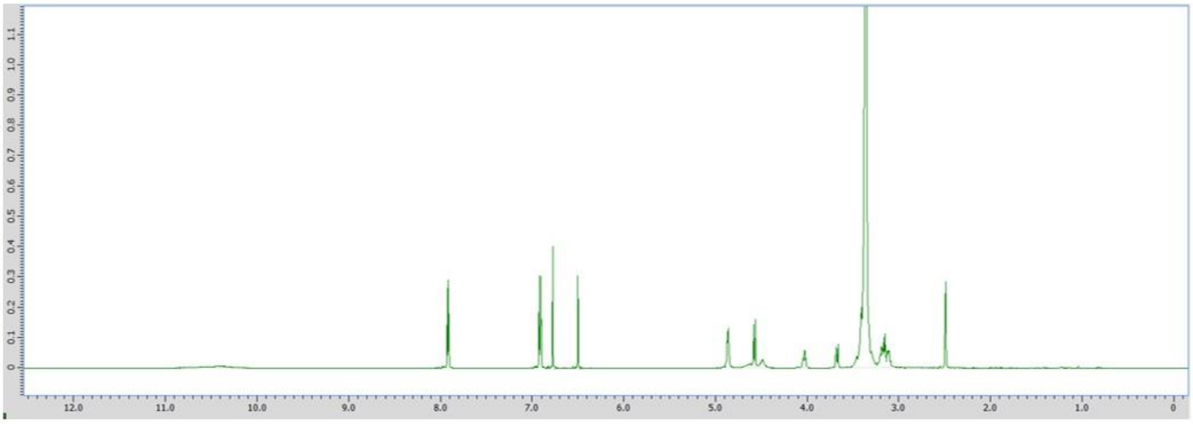

Figure S13. ^{1}H-NMR (600MHz, DMSO-d_6) spectrum of compound **5**

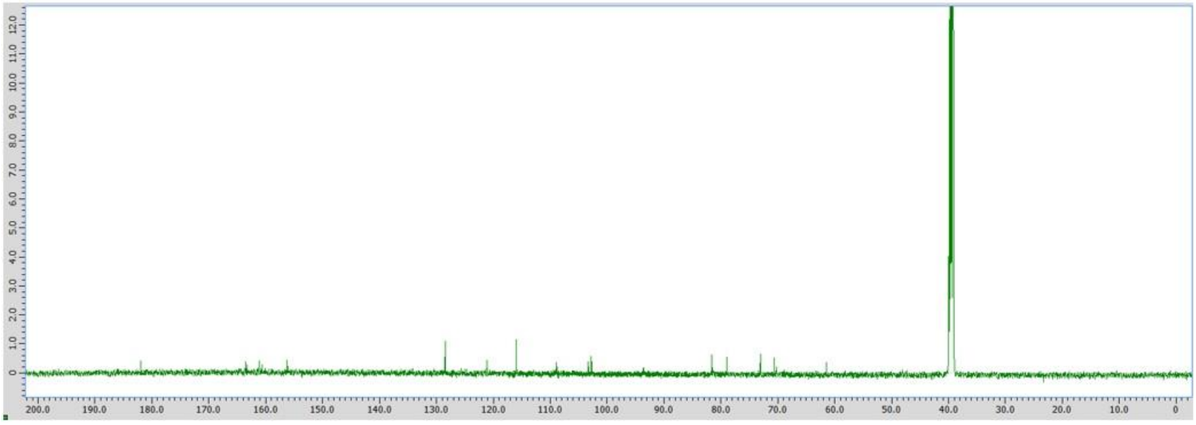

Figure S14. ^{13}C-NMR (150MHz, DMSO-d_6) spectrum of compound **5**

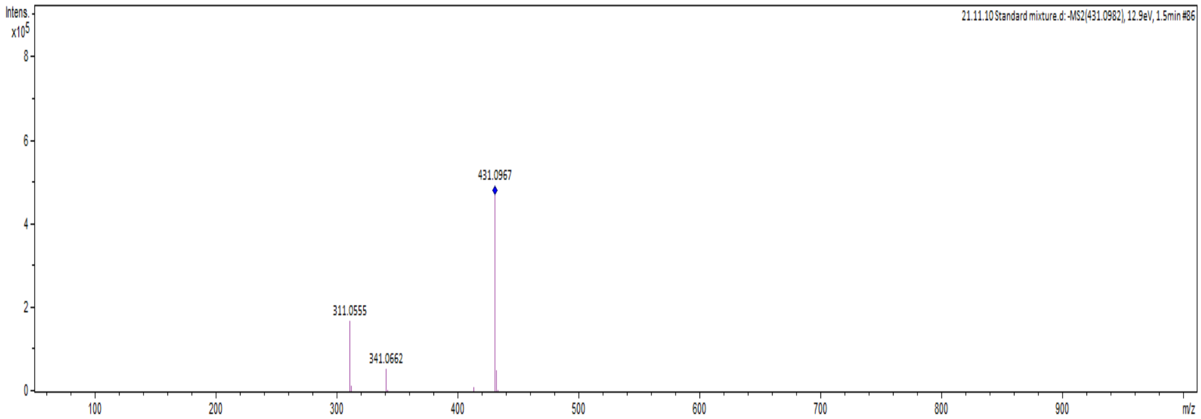

Figure S15. ESI/LTQ-Orbitrap-HRMS spectrum of compound **5** (negative mode)

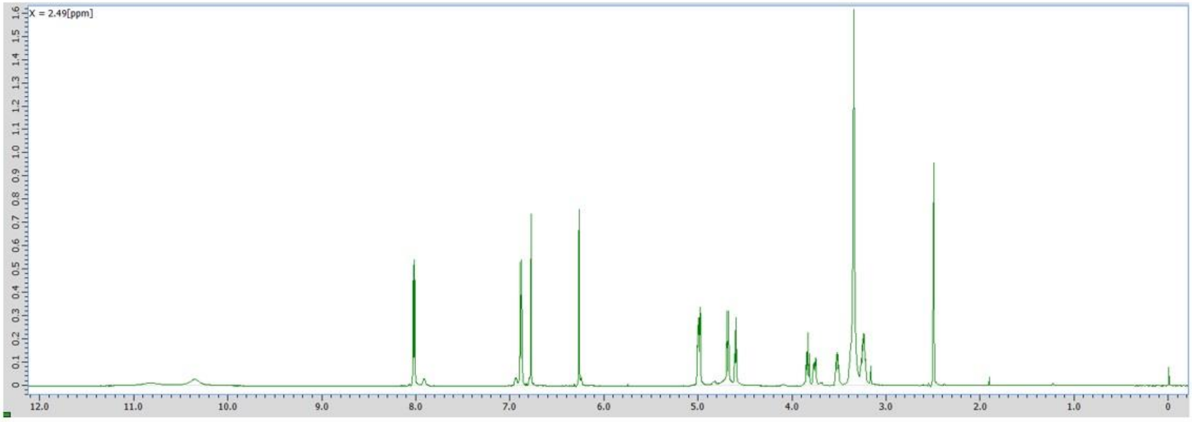

Figure S16. ^{1}H-NMR (600MHz, DMSO-*d*$_6$) spectrum of compound **6**

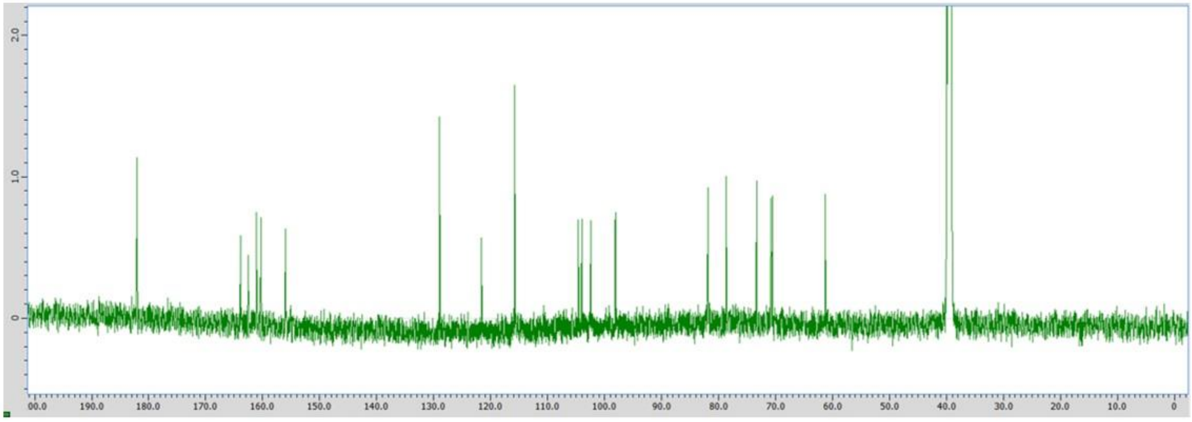

Figure S17. ^{13}C-NMR (150MHz, DMSO-*d*$_6$) spectrum of compound **6**

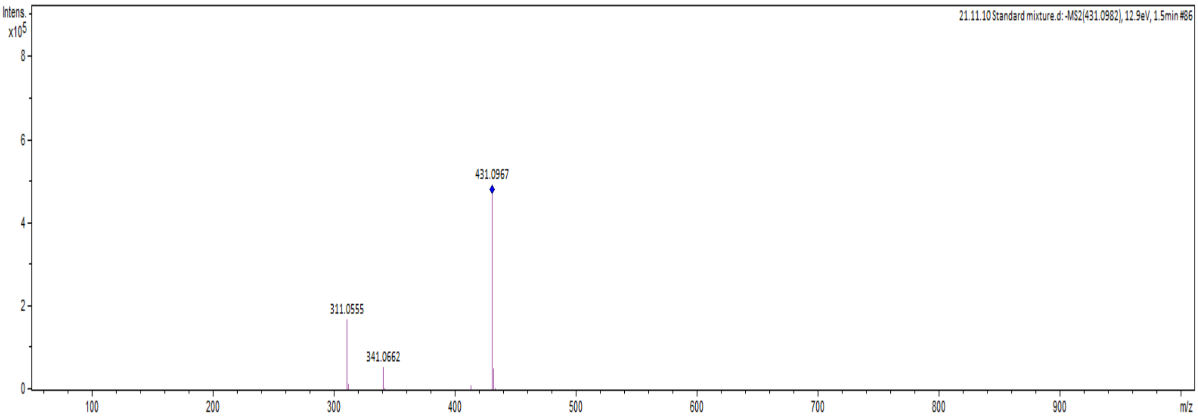

Figure S18. ESI/LTQ-Orbitrap-HRMS spectrum of compound **6** (negative mode)

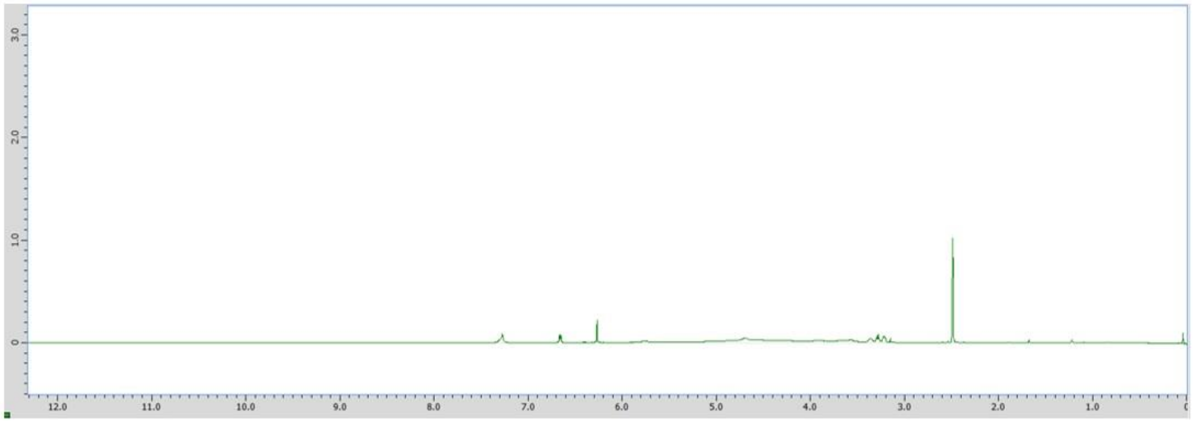

Figure S19. ^{1}H-NMR (600MHz, DMSO-d_6) spectrum of compound **7**

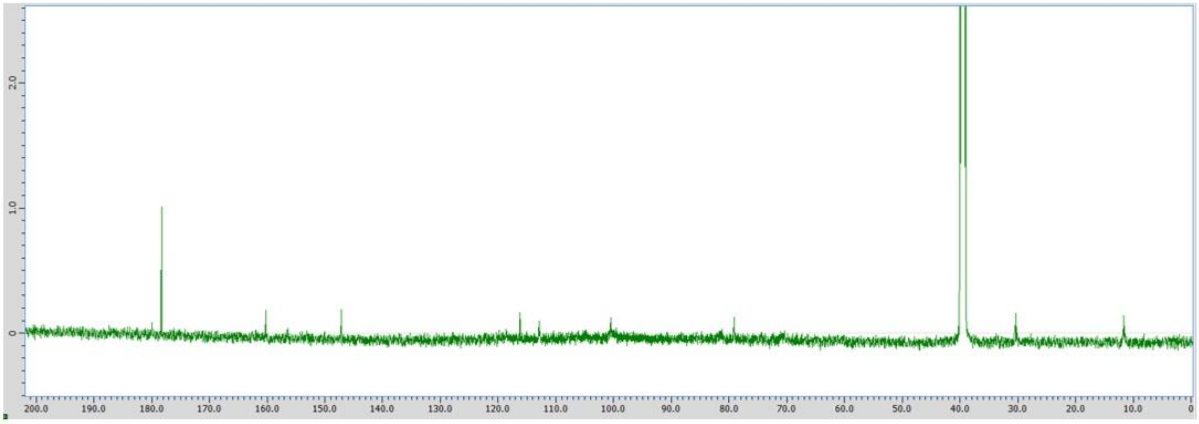

Figure S20. ^{13}C-NMR (150MHz, DMSO-d_6) spectrum of compound **7**

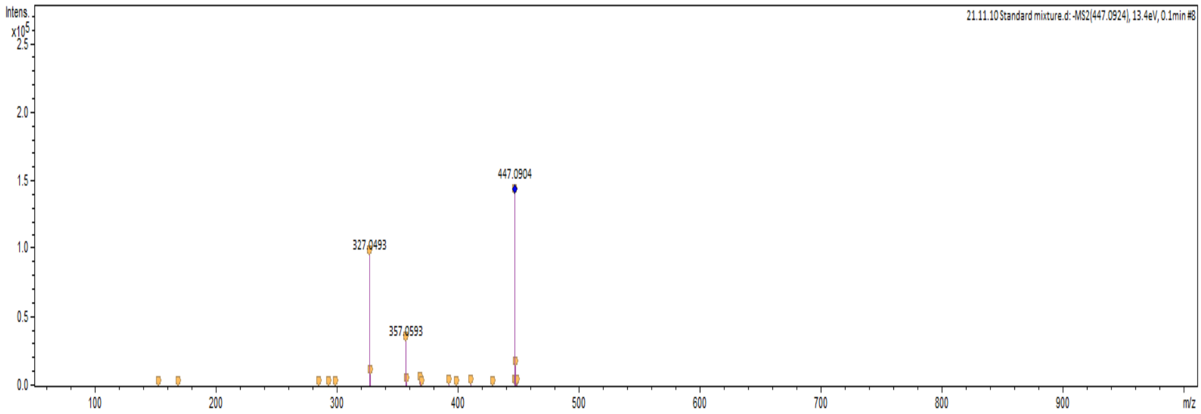

Figure S21. ESI/LTQ-Orbitrap-HRMS spectrum of compound **7** (negative mode)

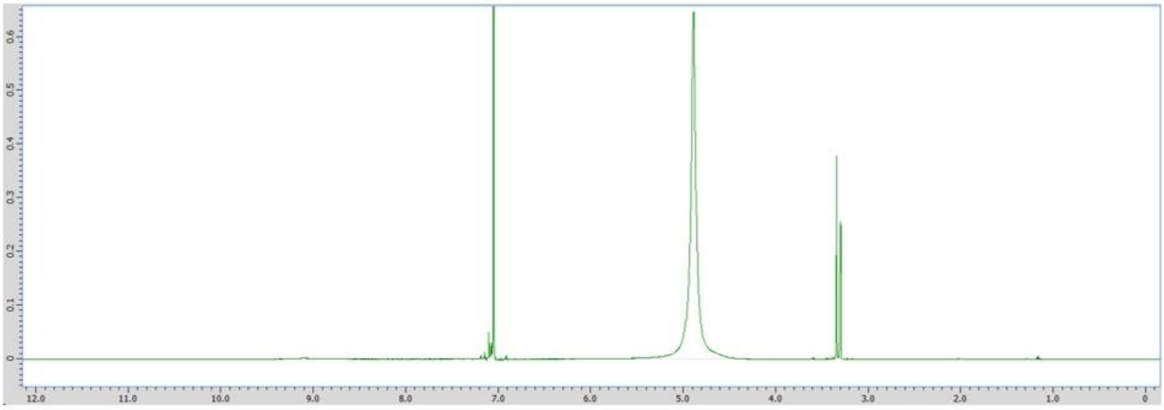

Figure S22. ^{1}H-NMR (600MHz, CD_3OD-*d*$_4$) spectrum of compound **8**

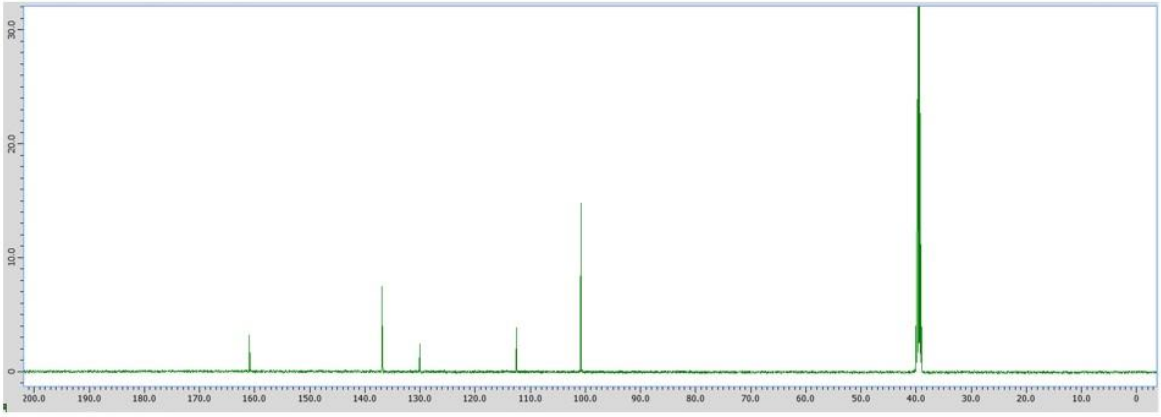

Figure S23. ^{13}C-NMR (150MHz, CD_3OD-*d*$_4$) spectrum of compound **8**

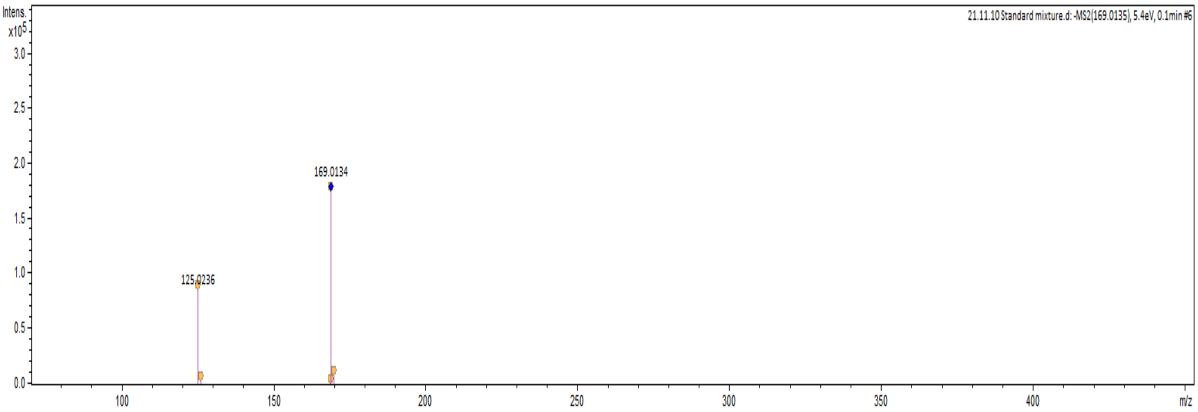

Figure S24. ESI/LTQ-Orbitrap-HRMS spectrum of compound **8** (negative mode)

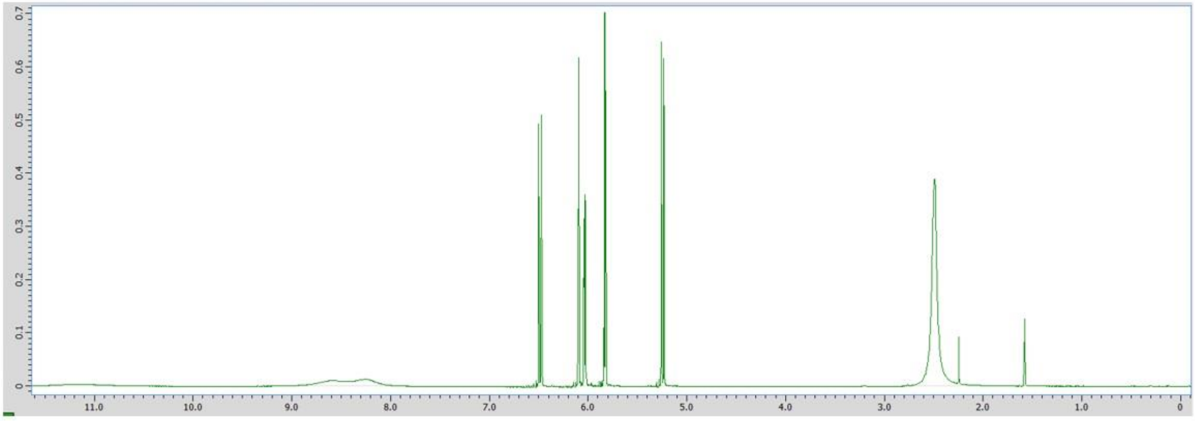

Figure S25. ^{1}H-NMR (600MHz, DMSO-d_6) spectrum of compound **9**

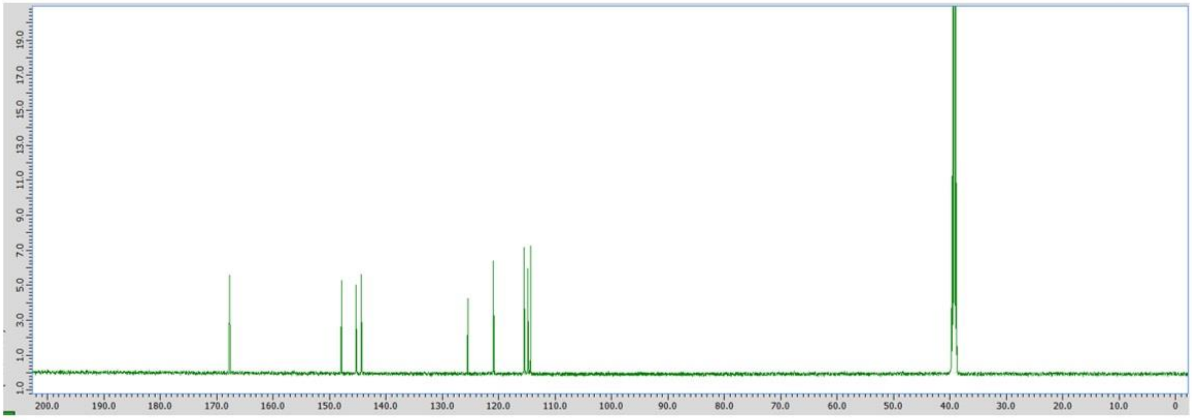

Figure S26. ^{13}C-NMR (150MHz, DMSO-d_6) spectrum of compound **9**

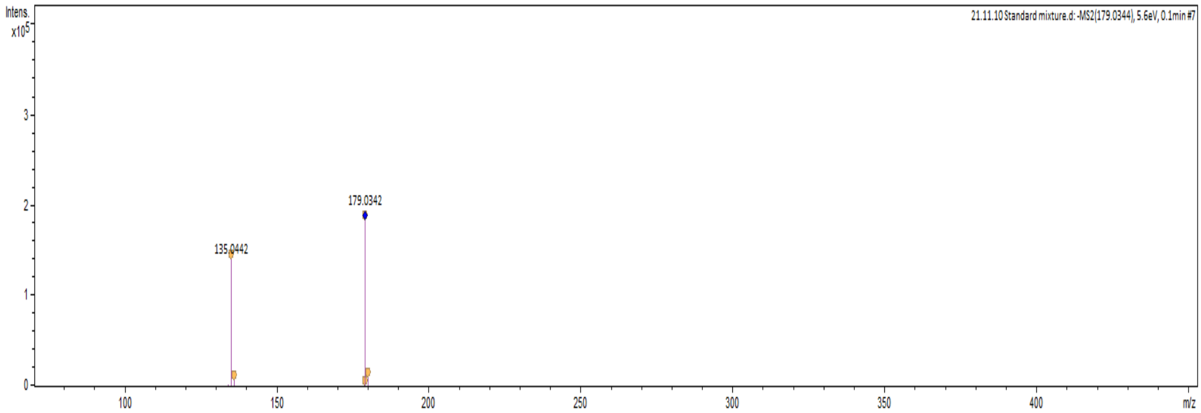

Figure S27. ESI/LTQ-Orbitrap-HRMS spectrum of compound **9** (negative mode)

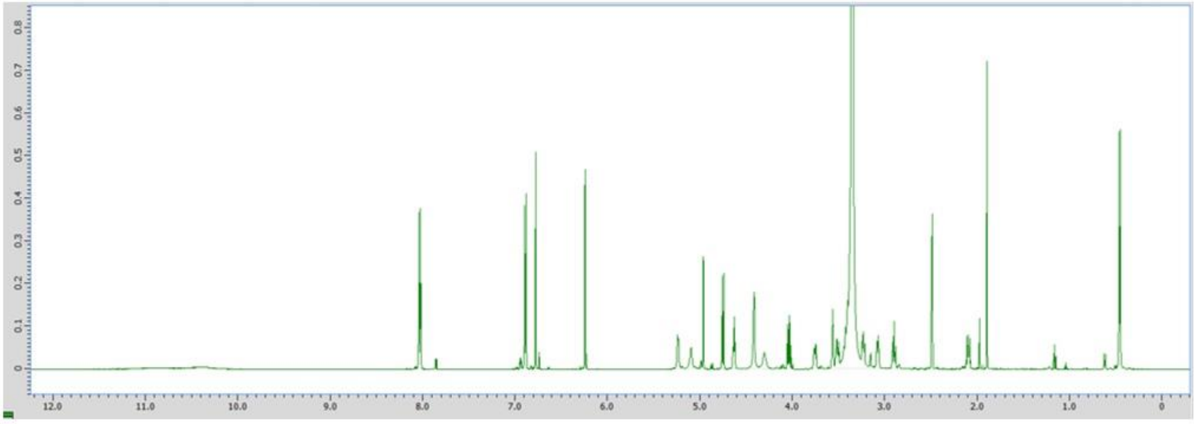

Figure S28. ^{1}H-NMR (600MHz, DMSO-d_6) spectrum of compound **10**

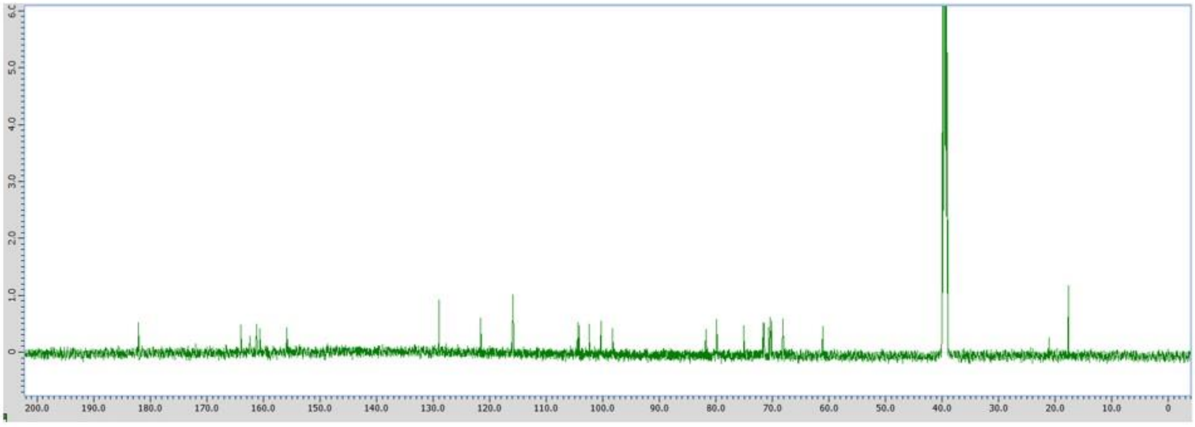

Figure S29. ^{13}C-NMR (150MHz, DMSO-d_6) spectrum of compound **10**

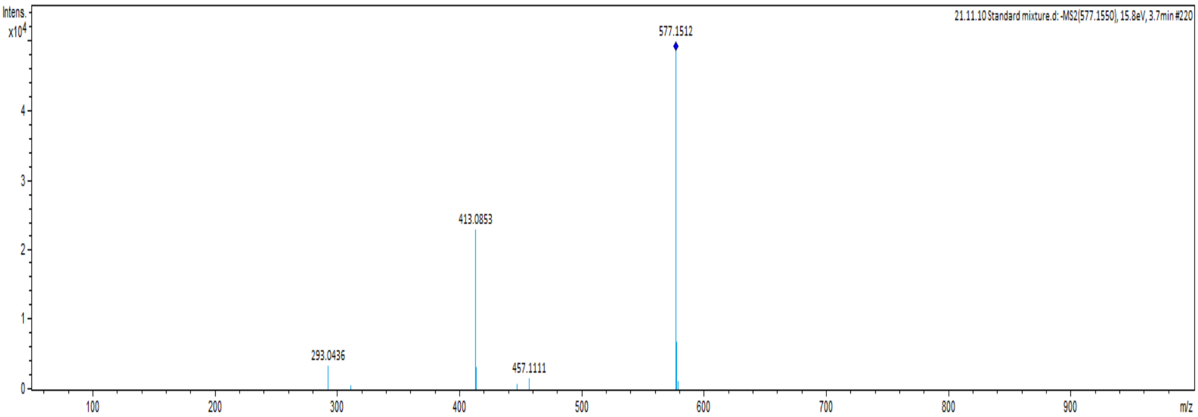

Figure S30. ESI/LTQ-Orbitrap-HRMS spectrum of compound **10** (negative mode)

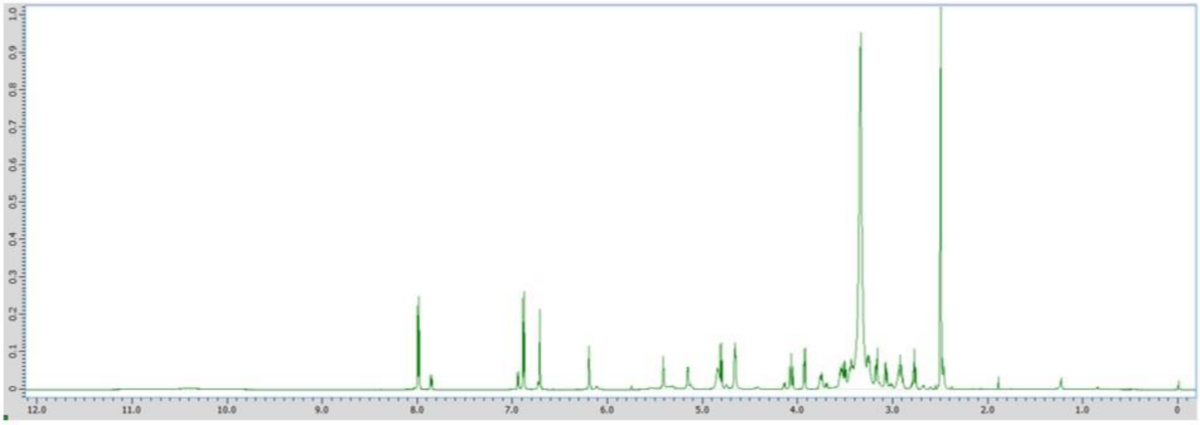

Figure S31. ^{1}H-NMR (600MHz, DMSO-d_6) spectrum of compound **11**

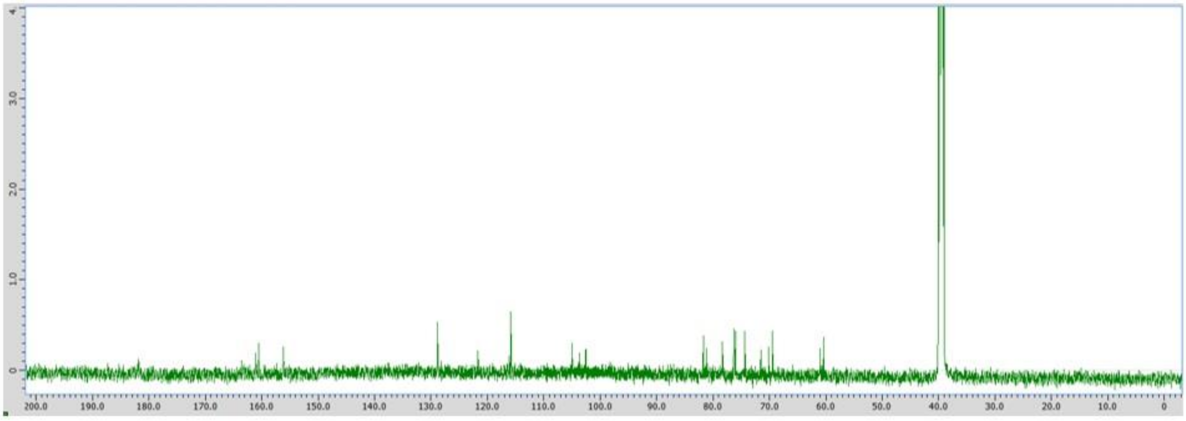

Figure S32. ^{13}C-NMR (150MHz, DMSO-d_6) spectrum of compound **11**

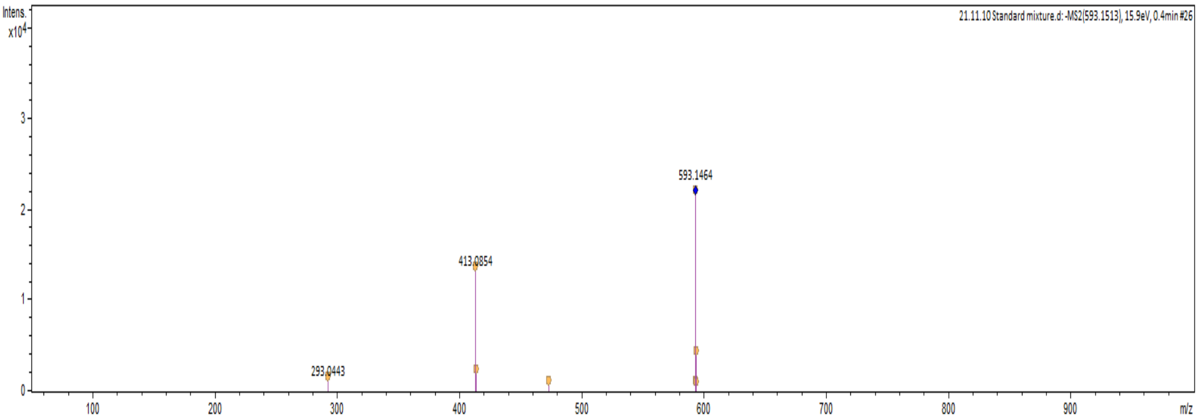

Figure S33. ESI/LTQ-Orbitrap-HRMS spectrum of compound **11** (negative mode)

Table S1. Recovery of compounds (1- 11) for three different spiked concentrations (5, 25, and 50 μg/mL).

**Compound**	**Spiked concentration (μg/mL)**	**Recovery rate (%)**
1	5	95.42
	25	95.03
	50	99.48
2	5	98.23
	25	100.26
	50	103.78
3	5	93.69
	25	97.04
	50	98.98
4	5	102.83
	25	101.74
	50	103.27
5	5	95.99
	25	94.64
	50	96.33
6	5	98.68
	25	97.92
	50	98.12
7	5	97.70
	25	98.00
	50	95.69
8	5	103.89
	25	98.26
	50	98.75
9	5	104.51
	25	97.22
	50	96.82
10	5	99.65
	25	98.34
	50	102.05
11	5	95.79
	25	95.06
	50	94.80

Table S2. Robustness of compounds (1-11) under various analytical conditions (flow rate, column temp., and solvent modifier concentration).

Compound	Condition		Capacity Factor (K')	Resolution (Rs)	Theoretical plate (N)
1	Flow rate (mL/min)	0.9	3.83	9.25	14815.44
		1.0	4.02	9.22	11952.50
		1.1	5.16	8.84	9082.20
	Column temp. (℃)	20	4.11	8.89	11097.75
		25	4.02	9.22	11952.50
		30	3.75	8.14	10291.55
	Acetic acid (%)	0.05	4.00	8.78	12133.57
		0.1	4.02	9.22	11952.50
		0.15	3.99	8.00	10153.73
2	Flow rate (mL/min)	0.9	2.26	4.58	5319.16
		1.0	2.35	4.69	5679.13
		1.1	3.04	5.09	5312.66
	Column temp. (℃)	20	2.41	5.15	5245.97
		25	2.35	4.69	5679.13
		30	2.16	4.03	3817.59
	Acetic acid (%)	0.05	2.33	4.03	4502.03
		0.1	2.35	4.69	5679.13
		0.15	2.32	4.07	3610.35
3	Flow rate (mL/min)	0.9	6.21	0.84	15951.69
		1.0	6.18	0.63	16486.56
		1.1	5.90	0.67	12203.82
	Column temp. (℃)	20	5.81	1.12	16092.73
		25	6.18	0.63	16486.56
		30	5.86	0.46	12356.34
	Acetic acid (%)	0.05	6.12	0.76	14953.11
		0.1	6.18	0.63	16486.56
		0.15	6.07	0.83	17257.66
4	Flow rate (mL/min)	0.9	1.50	2.78	4255.21
		1.0	1.54	2.62	3739.36
		1.1	2.00	2.94	4231.45
	Column temp. (℃)	20	1.54	3.10	4697.52
		25	1.54	2.62	3739.36
		30	1.45	3.42	4399.98
	Acetic acid (%)	0.05	1.51	2.71	2283.50
		0.1	1.54	2.62	3739.36
		0.15	1.49	2.71	2837.21
5	Flow rate (mL/min)	0.9	5.99	0.86	19651.73
		1.0	6.53	1.69	25316.86
		1.1	7.44	1.90	24017.11
	Column temp. (℃)	20	6.52	1.44	21431.01
		25	6.53	1.69	25316.86
		30	6.30	1.69	22284.73
	Acetic acid (%)	0.05	6.42	1.29	17793.80
		0.1	6.53	1.69	25316.86
		0.15	6.38	1.46	21821.53
6	Flow rate	0.9	5.24	2.12	18254.12

	(mL/min)	1.0	5.72	2.32	17529.36
		1.1	5.38	2.28	18952.07
	Column temp. (℃)	20	5.97	2.41	16854.21
		25	5.89	2.22	15478.72
		30	5.81	2.61	14998.34
	Acetic acid (%)	0.05	5.63	2.08	16952.49
		0.1	5.66	2.11	17254.77
		0.15	5.59	1.97	17970.20
7	Flow rate (mL/min)	0.9	5.18	3.83	44078.09
		1.0	5.62	4.93	36975.44
		1.1	5.44	5.55	45708.00
	Column temp. (℃)	20	5.63	4.29	40794.16
		25	5.62	4.93	36975.44
		30	5.38	4.31	32303.30
	Acetic acid (%)	0.05	5.53	4.45	40059.85
		0.1	5.62	4.93	36975.44
		0.15	5.49	4.48	36492.46
8	Flow rate (mL/min)	0.9	0.93	-	914.75
		1.0	0.96	-	1445.01
		1.1	1.34	-	1239.98
	Column temp. (℃)	20	0.98	-	1412.95
		25	0.96	-	1445.01
		30	0.86	-	1395.60
	Acetic acid (%)	0.05	0.96	-	1549.21
		0.1	0.96	-	1445.01
		0.15	0.94	-	1230.82
9	Flow rate (mL/min)	0.9	4.33	2.11	4380.67
		1.0	4.60	2.54	6619.45
		1.1	5.94	3.19	14680.06
	Column temp. (℃)	20	4.68	2.29	5445.13
		25	4.60	2.54	6619.45
		30	4.32	2.08	3645.67
	Acetic acid (%)	0.05	4.57	2.36	5489.64
		0.1	4.60	2.54	6619.45
		0.15	4.55	2.33	6153.84
10	Flow rate (mL/min)	0.9	5.54	1.21	19685.29
		1.0	6.05	1.62	23618.31
		1.1	5.74	0.97	19184.99
	Column temp. (℃)	20	6.02	1.39	18714.24
		25	6.05	1.62	23618.31
		30	5.76	1.21	25192.04
	Acetic acid (%)	0.05	5.93	1.03	17343.23
		0.1	6.05	1.62	23618.31
		0.15	5.89	1.49	17306.04
11	Flow rate (mL/min)	0.9	0.61	0.61	17552.67
		1.0	0.81	0.81	20352.39
		1.1	0.80	0.80	18181.19
	Column temp. (℃)	20	5.27	0.60	15417.80
		25	5.75	0.81	20352.39
		30	5.58	0.93	17306.53
	Acetic acid	0.05	0.61	5.64	15980.79

(%)	0.1	0.81	5.75	20352.39
	0.15	0.80	5.60	21229.22
